# DCIR-mediated inhibitory regulation of TLR7-MyD88 axis prevents autoimmune neuroinflammation

**DOI:** 10.1016/j.isci.2026.116094

**Published:** 2026-05-29

**Authors:** Atsushi Fujioka, Kenji Shimizu, Saki Nakayama, Dai Ueno, Yuki Chiba, Ichiro Nakashima, Juichi Fujimori, Yuko Shirota, Yoichiro Iwakura, Yasushi Muraki, Akira Nakamura, Tomonori Kaifu

**Affiliations:** 1 Division of Immunology, Faculty of Medicine, Tohoku Medical and Pharmaceutical University, 1-15-1, Fukumuro, Miyagino, Sendai 983-8536, Japan; 2 Laboratory of Molecular Immunology, Institute for Quantitative Biosciences, The University of Tokyo, 1-1-1, Yayoi, Bunkyo-ku, Tokyo 113-0032, Japan; 3 Division of Neurology, Faculty of Medicine, Tohoku Medical and Pharmaceutical University, 1-15-1, Fukumuro, Miyagino, Sendai 983-8536, Japan; 4 Division of Hematology and Rheumatology, Faculty of Medicine, Tohoku Medical and Pharmaceutical University, 1-15-1, Fukumuro, Miyagino, Sendai 983-8536, Japan; 5 Research Institute for Biomedical Sciences, Tokyo University of Science, 2669 Yamazaki, Noda, Chiba 278-0022, Japan; 6 Division of Infectious Disease and Immunology, Department of Microbiology, School of Medicine, Iwate Medical University, 1-1-1 Idaidori, Yahaba, Iwate 028-3694, Japan

**Keywords:** biological sciences, immunology, molecular biology

## Abstract

Encephalitogenic T cells are responsible for developing autoimmune diseases in the central nervous system (CNS), but the pathogenesis of CNS autoimmune diseases remains incompletely understood. Dendritic cell immunoreceptor (DCIR) is an inhibitory type of C-type lectin receptor that regulates the antigen presentation ability of DCs, and DCIR deficiency exacerbates autoimmune diseases. Here, we demonstrated that DCIR deficiency induced spontaneous development of experimental autoimmune encephalomyelitis (EAE)-like encephalomyelitis in 2D2 TCR transgenic mice (2D2Tg) expressing a myelin oligodendrocyte glycoprotein (MOG)-specific T cell receptor. 2D2Tg*Dcir*^−/−^*Myd88*^−/−^ mice negated the spontaneous development of EAE-like encephalomyelitis. Moreover, 2D2Tg*Dcir*^−/−^*Tlr7*^−/−^ mice developed less EAE-like encephalomyelitis. Interestingly, an endogenous ligand of TLR7, U11snRNA, was detected in the sera of 2D2Tg*Dcir*^−/−^ mice and the patients of multiple sclerosis, indicating excessive DC activation by TLR7-mediated signaling in 2D2Tg*Dcir*^−/−^ mice and patients with a neuronal autoimmune disease. Thus, defective DCIR overstimulates autoreactive T cells by strengthening TLR7-mediated responses, thereby exacerbating TLR7-MyD88-mediated autoimmune diseases.

## Highlights

• 2D2Tg mice with DCIR deficiency develop a spontaneous neuronal autoimmune disease
• DCIR negatively regulates TLR7-MyD88 signal pathways
• U11snRNA, a ligand for TLR7, is a potential pathogenic factor in neuroinflammation
• Serum U11snRNA is related to chronic inflammation of multiple sclerosis

## Graphical abstract

**Figure:**
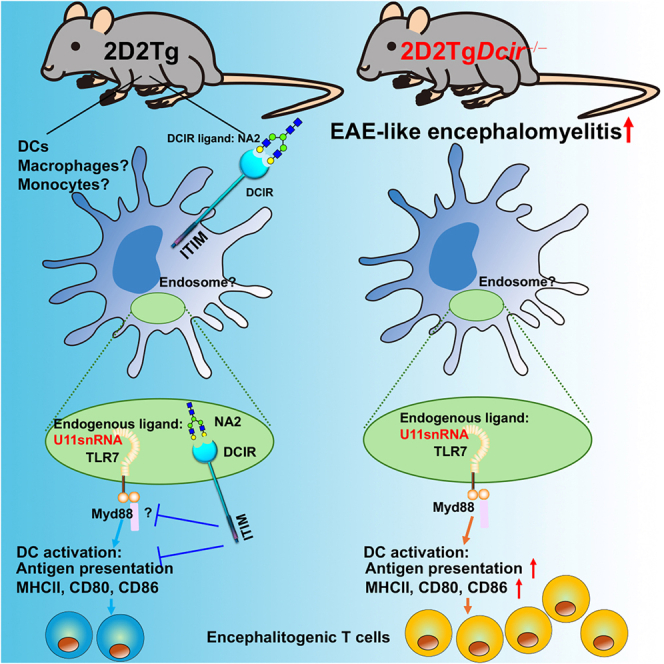

## Introduction

More than 2.8 million people worldwide are estimated to have multiple sclerosis (MS),^1^^,^^2^ a chronic inflammatory disease of the brain and spinal cord. MS is characterized by lymphocytic infiltration and neuroinflammation, leading to concomitant damage of axons and neurons that cause severe physical disability. The precise cause of MS remains elusive, but the disease is considered to be an autoimmune disease caused by encephalitogenic T cells.^3^^,^^4^ Experimental autoimmune encephalomyelitis (EAE) has served as an animal model for MS, which is induced by immunization of various myelin antigens, including myelin basic protein (MBP), proteolipid protein (PLP), and myelin oligodendrocyte glycoprotein (MOG), indicating that self-antigens in the CNS are involved in the pathogenesis of MS. EAE is an essential tool not only to evaluate the preclinical effect of drugs but also to understand the pathogenesis of MS. However, EAE has several drawbacks, such as the harsh disease induction caused by adjuvants, which should be considered when interpreting MS results from EAE.

Dendritic cell immunoreceptor (DCIR; gene symbol, *Clec4a2*) is a C-type lectin receptor (CLR), which was identified by searching the human nucleotide database with a motif (SCYWFSH) shared between the carbohydrate recognition domain (CRD) of the hepatic ASGPRs (asialoglycoprotein receptors) and the macrophage lectin.^5^ DCIR possesses a CRD in the extracellular region, containing an EPS (glutamic acid-proline-serine) motif similar to the EPN (glutamic acid-proline-asparagine) motif that binds mannose, glucose, GlcNAc, and fucose. We identified asialo-biantennary N-glycan (NA2) as a functional endogenous ligand for DCIR.^6^ DCIR possesses a canonical immunoreceptor tyrosine-based inhibitory motif (ITIM) in its intracellular region, providing docking sites for phosphatases SHP-1 and SHP-2.^7^ The structural characteristics indicate that DCIR is an inhibitory-type receptor that suppresses cellular activation responses.

*Dcir*^−/−^ mice spontaneously develop autoimmune-like and bone metabolic diseases with age.^8^^,^^9^ Autoimmune enthesitis and sialadenitis were observed in the ankle joints and salivary glands, and ankylotic changes in the ankle joints developed due to fibrocartilage proliferation and heterotopic ossification within the joints. Interestingly, *Dcir*^−/−^ mice increased femoral bone mass. *Dcir*^−/−^ mice had increased IFN-γ-producing T cells, and the antigen presentation ability in *Dcir*^−/−^ DCs was upregulated to induce higher IFN-γ-producing T cells. IFN-γ is an accelerator for excess chondrogenesis and bone formation.^9^ Moreover, *Dcir*^−/−^ mice are hyperresponsive to EAE, an animal model for MS, and the administration of neuraminidase, which increases the expression of asialo-DCIR ligand, ameliorates EAE clinical score in a DCIR-dependent manner.^6^^,^^10^ We also found that DCIR deficiency increased gene expression associated with lipid and glycogen metabolism and DCIR down-regulated M-CSF and RANKL signaling.^11^ Thus, DCIR is a critical regulatory CLR in maintaining the homeostasis of the immune system and bone metabolism, and the defective DCIR exacerbates bone and autoimmune diseases.

Toll-like receptors (TLRs) are pattern-recognition receptors that play a crucial role in detecting pathogen invasion. TLRs and a common signal adapter protein, MyD88, are implicated in the pathogenesis of EAE. MyD88 is a signal adapter protein essential for the development of EAE because *Myd88*^−/−^ mice were not susceptible to EAE.^12^ TLR7 utilizes MyD88 as a signal protein complex and *Tlr7*^−/−^ mice ameliorated EAE clinical scores due to enhanced differentiation of Treg cells. Moreover, IFN-β secreted from human DCs via TLR7 signaling inhibits cytokine production, which is responsible for Th17 differentiation.^13^ TLR7 is an RNA-sensing receptor that recognizes single-stranded RNA derived from the virus, and the expression amplification and chronic activation of TLR7 are involved in immune-related diseases.^14^^,^^15^ Negishi et al. found that U11 small nuclear RNA (U11snRNA) is an endogenous TLR7 agonistic ligand, and U11snRNA levels are elevated in the sera of patients with rheumatoid arthritis (RA) and systemic lupus erythematosus (SLE). Thus, the TLR7-MyD88 axis is one of the important causative factors for EAE.

2D2 TCR transgenic mice (2D2Tg) expressed a TCR specific for the MOG_35-55_ peptide and developed optic neuritis without exhibiting limb paralysis.^16^ However, the incidence of EAE-like encephalomyelitis developed spontaneously was low in 2D2Tg mice, indicating that the presence of potential autoreactive T cells is insufficient for the spontaneous development of EAE-like encephalomyelitis. In this study, we have revealed that DCIR deficiency in 2D2Tg mice (2D2Tg*Dcir*^−/−^ mice) remarkably increased the spontaneous development of EAE-like encephalomyelitis compared to 2D2Tg mice. DCIR deficiency had no effect on the development and compartmentalization of T cells. However, 2D2Tg*Dcir*^−/−^ mice had a higher incidence of EAE-like encephalomyelitis with increased mortality. Splenocytes of 2D2Tg*Dcir*^−/−^ mice produced higher amounts of IFN-γ and IL-17A than those of 2D2Tg mice in *in vitro* response to MOG_35-55_. MyD88 deficiency in 2D2Tg*Dcir*^−/−^ mice negated the increased spontaneous development of EAE-like encephalomyelitis, and *Dcir*^−/−^ DCs produced higher pro-inflammatory cytokines upon stimulation of imiquimod (IMQ), an agonist of TLR7. TLR7 deficiency in 2D2Tg*Dcir*^−/−^ mice showed comparable spontaneous development of EAE-like encephalomyelitis to that in 2D2Tg mice. Importantly, we detected serum U11snRNA, an endogenous agonist of TLR7, in 2D2*TgDcir*^−/−^ mice, and higher levels of U11snRNA in the sera of MS patients. Therefore, defective DCIR increases the risk of developing CNS autoimmune diseases by activating the TLR7-signaling pathway, thereby inducing autoreactive T cells. Our study highlights the regulatory role of DCIR in the TLR7-MyD88 axis, which prevents autoimmune neuroinflammation.

## Results

### DCIR is dispensable for T cell differentiation and compartmentalization

DCIR is expressed in myeloid cells, such as dendritic cells (DCs), macrophages, and osteoclasts. *Dcir*^−/−^ mice spontaneously develop autoimmune-like symptoms and are more susceptible to EAE than wild-type mice (C57BL/6J mice).^6^^,^^8^^,^^10^ These findings demonstrate that DCIR is one of the regulatory receptors that maintain immune system homeostasis, and dysfunction of DCIR leads to the exacerbation of autoimmune diseases. To understand a pathogenic factor inducing spontaneous EAE in 2D2Tg mice, we generated 2D2*Dcir*^−/−^ mice by crossing 2D2Tg mice with *Dcir*^−/−^ mice.

To evaluate the effect of DCIR deficiency on the compartment of T cells, we examined the cellular distribution of CD4^+^ T cells with TCR composed of Vα3.2 and Vβ11 chains^16^ in the thymus, spleen, and lymph node. The distribution of CD4^+^ and CD8^+^ cells of 2D2Tg*Dcir*^−/−^ mice in the thymus, spleen, and lymph node was comparable to those of 2D2Tg mice (Figures S1A–S1C). CD4^+^ thymocytes, splenocytes, and lymphocytes were dominant in 2D2 TCR transgenic mice, compared with nontransgenic mice (*Dcir*^−/−^ mice) (Figures S1D–S1F). The CD4^+^ cells of 2D2Tg and 2D2Tg*Dcir*^−/−^ mice dominantly expressed TCR composed of Vα3.2 and Vβ11 chains (Figures S1A–S1F).

### DCIR deficiency leads to the spontaneous development of EAE-like encephalomyelitis

**Figure 1:**
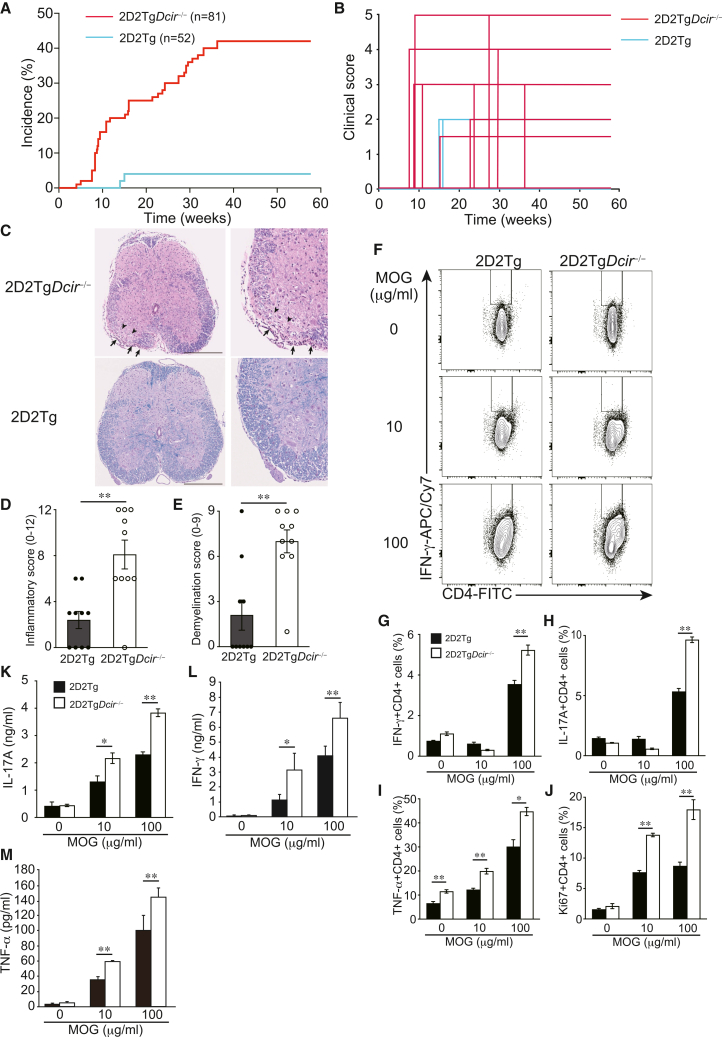
DCIR deficiency induces the spontaneous development of autoimmune encephalomyelitis and T cell responses are increased after MOG restimulation under steady-state conditions in 2D2Tg*Dcir*^−/−^ mice (A) 2D2Tg mice (*n* = 52) and 2D2Tg*Dcir*^−/−^ mice (*n* = 81) were monitored for 58 weeks. The clinical disease score was observed once per week. The incidence was calculated using all mice regardless of disease development. (B) Disease severity of spontaneous development of EAE-like encephalomyelitis in 2D2Tg mice (sky blue) and 2D2Tg*Dcir*^−/−^ mice (red). (C) Histology of spinal cords in affected 2D2Tg*Dcir*^−/−^ mice (upper) and unaffected 2D2Tg mice (lower). The sections of the spinal cords at 20 weeks were stained with H&E or LFB. Representative images are shown (scale bars, 250 μm). Arrows indicate infiltration of inflammatory cells into the spinal cords, and arrowheads show demyelination of white matter. (D and E) Inflammation and demyelination scores were assessed with histological examination of H&E and LFB staining (three spinal cord sections per mouse were evaluated). The bars show the mean ± SEM. Statistical significance was evaluated using a Mann-Whitney U test (∗∗, *p* < 0.01, ∗, *p* < 0.05). (F) Splenocytes recall responses to MOG_35-55_ peptide. Splenocytes were harvested from 2D2Tg mice and 2D2Tg*Dcir*^−/−^ mice and stimulated at the indicated concentration of MOG_35-55_ peptide for 3 days, following intracellular staining of splenocytes gated on CD4^+^ cells. The numeric digits in dot plots show the cell proportion inside a rectangle. (G–J) Histogram analysis of cytokine-positive cells in (F). (K–M) Cytokine production from splenocytes in response to MOG_35-55_ peptide. The cytokine concentrations were determined with ELISA (k; IL-17A, l; IFN-γ, m; TNF-α). Black bar: 2D2Tg mice, white bar: 2D2Tg*Dcir*^−/−^ mice. The bars show mean ± SD of triplicate wells and the data are representative of two independent experiments (G–M). ∗∗, *p* < 0.01, ∗, *p* < 0.05.

We monitored the development of EAE in 2D2Tg and 2D2Tg*Dcir*^−/−^ mice under specific pathogen-free (SPF) conditions. The incidence of EAE-like encephalomyelitis that developed spontaneously in 2D2Tg mice was 3.8% (2/52) under our breeding conditions, with no mortality, consistent with the incidence reported in the Bettelli study^16^ (Figures 1A and 1B; Table 1). The incidence of spontaneous development of EAE-like encephalomyelitis in 2D2Tg*Dcir*^−/−^ mice, however, increased by 42% (34/81) with 16% (13/81) mortality (Figures 1A and 1B; Table 1). The apparent differences of the maximum clinical score (2D2Tg*Dcir*^−/−^ mice and 2D2Tg mice are 3.2 ± 1.7 vs. 2.5 ± 0.7) and disease onset (2D2Tg*Dcir*^−/−^ mice and 2D2Tg mice is 19.5 ± 11.8 weeks vs. 15.5 ± 4.9 weeks) weren’t observed, and the EAE-like encephalomyelitis symptoms appeared between 2 and 5 months of age (Table 1). A series of tissue sections from spinal cords was stained with H&E and Luxol Fast Blue (LFB) to examine cell infiltration and demyelination. H&E staining revealed that leukocyte infiltration was observed below the meninges and inside the parenchyma, and the inflammation score in 2D2Tg*Dcir*^−/−^ mice was higher than that in 2D2Tg mice (Figures 1C and 1D). In addition, LFB staining showed that demyelination in the white matter, with a weaker pale blue area, was increased in 2D2Tg*Dcir*^−/−^ mice (Figures 1C and 1E). These data demonstrated that DCIR deficiency in 2D2Tg mice facilitates EAE development under SPF conditions.

**Table 1:** Spontaneous development of EAE-like symptoms in 2D2Tg*Dcir*^−/−^ mice

Strain	incidence	mortality	maximum clinical score	weeks of onset
2D2Tg*Dcir*^−/−^	34/81(42%)^a^	13/81(16%)	3.2 ± 1.7 (affected mice)	19.5 ± 11.8
2D2Tg 2/5	2(3.8%)	0/52(0%)	2.5 ± 0.7 (affected mice)	15.5 ± 4.9

Data are presented as means ± SD.

a *p* < 0.01, chi-square test.

Chronic inflammation induced by the activation of encephalitogenic T cells in the CNS accounts for MS and EAE, characterized by demyelination damage. We hypothesized that DCIR deficiency would promote T cell cytokine production in response to MOG_35-55_. Splenocytes were collected from 2D2Tg mice and 2D2Tg*Dcir*^−/−^ mice and were stimulated *in vitro* with different concentrations of MOG_35-55_ peptide for 3 days. Flow cytometer (FCM) analysis showed that IFN-γ-, IL-17A-, and TNF-α-positive T cells were significantly increased in 2D2Tg*Dcir*^−/−^ mice, and T cells positive for Ki67, a proliferation marker, were also higher in 2D2Tg*Dcir*^−/−^ mice than 2D2Tg mice (Figures 1F–1J). The amounts of cytokines from splenocytes stimulated by MOG_35-55_ were determined with ELISA, and we detected a significant increase of IL-17A,IFN-γ, and TNF-α in response to MOG_35-55_ (Figures 1K–1M). These data showed that DCIR deficiency enhances the antigen-specific activation of T cells, suggesting that, as T cells don’t express DCIR, DCs are responsible for the excessive responses of T cells in 2D2Tg*Dcir*^−/−^ mice.

### Inflammation evoked by PTX administration causes severe EAE-like encephalomyelitis in 2D2Tg*Dcir*^−/−^ mice

**Figure 2:**
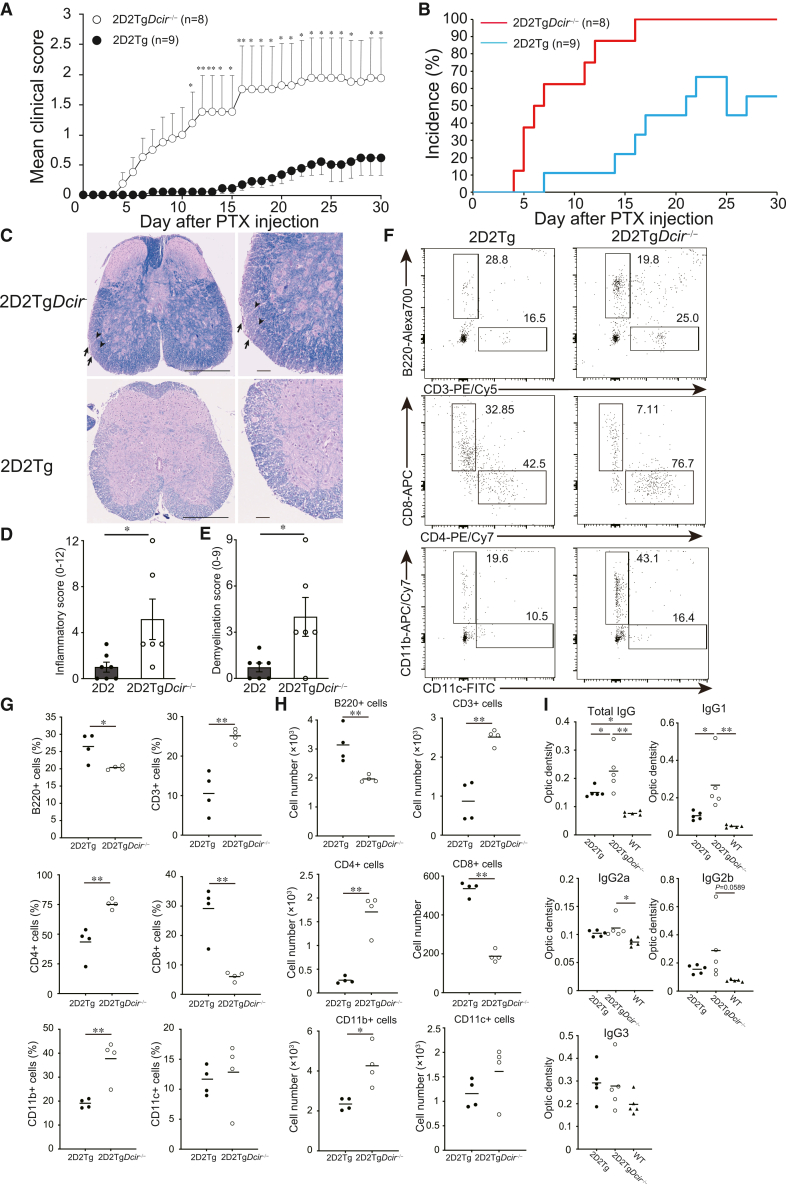
PTX administration caused severe EAE-like encephalomyelitis in 2D2Tg*Dcir*^−/−^ mice (A) EAE-like encephalomyelitis development of 2D2Tg mice and 2D2Tg*Dcir*^−/−^ mice after PTX administration. 2D2Tg mice (*n* = 9) and 2D2Tg*Dcir*^−/−^ mice (*n* = 8) were administered PTX twice, at day 0 and day 2, and monitored for 30 days. Statistical significance was evaluated using the Mann-Whitney U test (∗, *p* < 0.05). (B) Incidence of EAE-like encephalomyelitis after PTX administration in 2D2Tg mice (sky blue) and 2D2Tg*Dcir*^−/−^ mice (red). The data were representative of two independent experiments. (C) Histology of spinal cords in 2D2Tg*Dcir*^−/−^ mice with PTX-induced EAE-like encephalomyelitis. The sections of the spinal cords at 30 days after PTX injection were double-stained with H&E and LFB. Representative images are shown (scale bars, 250 μm in low-power fields and 50 μm in high-power fields). Arrows indicate infiltration of inflammatory cells into the spinal cords, and arrowheads show demyelination of white matter. (D and E) Inflammation and demyelination scores were assessed through histological examination of the H&E and LFB staining (three spinal cord sections per mouse were evaluated). Each symbol represents an individual mouse, and the bars show the mean ± SEM. Statistical significance was assessed using the Mann-Whitney U test (∗, *p* < 0.05). (F) Infiltrated cells in the spinal cords at day 15 after PTX administration. The cell proportions of T cells and myeloid cells from 2D2Tg*Dcir*^−/−^ and 2D2Tg mice are shown. (G) Plot analyses of cell distribution for F. (H) The cell numbers of infiltrated cells in the spinal cords for (F). Each symbol represents an individual mouse, and a horizontal bar indicates the mean of each score. The data are representative of two independent experiments (G and H). (I) Anti-MOG_35-55_ antibody titers at 30 days after PTX administration. Sera were collected from 2D2Tg*Dcir*^−/−^ mice (*n* = 5, open circle), 2D2Tg mice (*n* = 5, solid circle), and WT (*n* = 5, solid triangle) at day 30 after PTX injection and anti-MOG_35-55_ antibody titers were measured using ELISA. Each symbol represents an individual mouse, and a horizontal bar indicates the mean of each score. Statistical significances were evaluated using one-way ANOVA with Dunnett’s post hoc test (∗∗, *p* < 0.01, ∗, *p* < 0.05). This assay was performed with sera from two independent experiments.

EAE is induced in mice immunized with MOG_35-55_ peptide and administered with pertussis toxin (PTX), which is used as an adjuvant to enhance EAE. Bettelli et al. have reported that 2D2Tg mice possess autoreactive myelin-specific T cells, and PTX stimulation triggered the development of EAE-like encephalomyelitis symptoms in 2D2Tg mice.^16^ We wondered whether 2D2Tg*Dcir*^−/−^ mice were more susceptible to EAE-like encephalomyelitis under the same conditions. The PTX administration induced earlier disease onset in 2D2Tg*Dcir*^−/−^ mice than in 2D2Tg mice, and incidence and maximal clinical score were increased in 2D2Tg*Dcir*^−/−^ mice, compared to 2D2Tg mice (Figures 2A and 2B; Table 2). H&E and LFB staining showed that the inflammatory score and demyelination were higher in 2D2Tg*Dcir*^−/−^ mice than in 2D2Tg mice (Figures 2C–2E). We analyzed lymphocyte and myeloid cell infiltration into the spinal cords 15 days after PTX administration using FCM. PTX injection showed higher frequencies and cell numbers of CD3^+^ cells in the spinal cords of 2D2Tg*Dcir*^−/−^ mice than those of 2D2Tg mice, but those of B220+ cells were higher in 2D2Tg mice (Figures 2F–2H). CD4^+^ T cells remarkably increased in 2D2Tg*Dcir*^−/−^ mice than in 2D2Tg mice, while CD8^+^ T cell infiltration was higher in 2D2Tg mice than in 2D2Tg*Dcir*^−/−^ mice (Figures 2F–2H). In addition, we measured the infiltration of myeloid cells, and CD11b+ cells were significantly increased in 2D2Tg*Dcir*^−/−^ mice compared to 2D2Tg mice but not CD11c+ cells (Figures 2F–2H).

**Table 2:** EAE-like encephalomyelitis in 2D2Tg*Dcir*^−/−^ mice after PTX administration

Strain	incidence	mortality	maximum clinical score	days of onset
2D2Tg*Dcir*^−/−^	8/8(100%)	2/8(16%)	2.1 ± 1.8 (affected mice)^a^	8.3 ± 4.0^b^
2D2Tg	6/9(66.7%)	0/9(0%)	0.7 ± 0.3 (affected mice)	16.2 ± 5.4

Data are presented as means ± SD.

a *p* < 0.05, Mann-Whitney U test.

b *p* < 0.01, two-tailed unpaired Student’s *t* test.

Moreover, we detected antibody titers against the MOG_35-55_ peptide in sera of PTX-induced mice. Total anti-MOG_35-55_ IgG antibody titer and the titers of IgG1 and IgG2a subtypes against MOG_35-55_ peptide were increased in 2D2Tg*Dcir*^−/−^ mice, compared to those of 2D2Tg mice. IgG2b tended to increase in 2D2Tg*Dcir*^−/−^ mice, but IgG3 was comparable (Figure 2H). These data showed that PTX administration-induced inflammation, without immunization with myelin antigen, exacerbated EAE in 2D2Tg*Dcir*^−/−^ mice by enhancing immune responses.

### MyD88 deficiency prevents the spontaneous development of EAE-like encephalomyelitis in 2D2Tg*Dcir*^−/−^ mice

**Figure 3:**
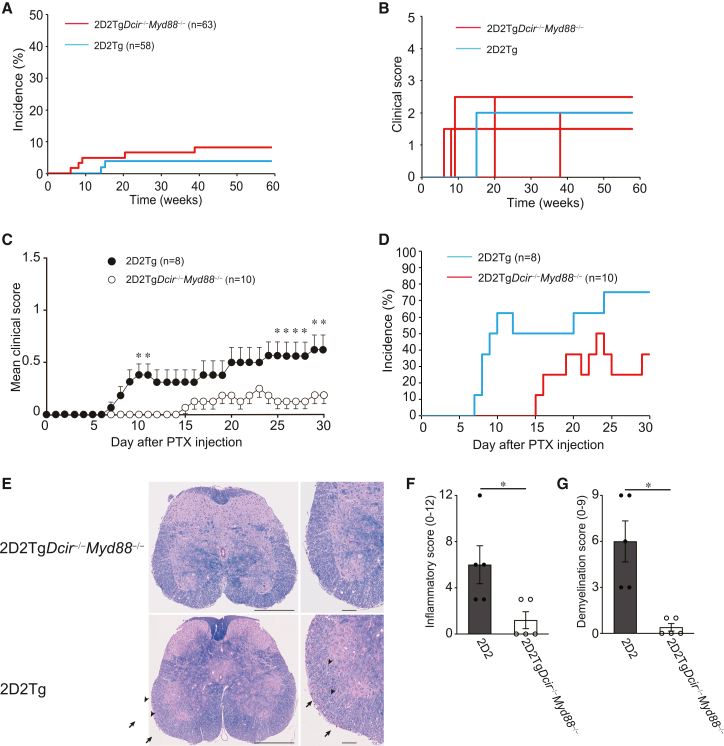
DCIR contributes to regulation in Myd88-mediated signaling (A) MyD88 deficiency negates the spontaneous development of EAE-like encephalomyelitis caused by DCIR deficiency. 2D2Tg mice (*n* = 52) and 2D2Tg*Dcir*^−/−^*Myd88*^−/−^mice (*n* = 63) were monitored for 58 weeks. The clinical disease score was observed once per week. (B) Disease severity of spontaneous development of EAE-like encephalomyelitis in 2D2Tg mice (sky blue) and 2D2Tg*Dcir*^−/−^*Myd88*^−/−^ mice (red). (C) EAE-like encephalomyelitis of 2D2Tg*Dcir*^−/−^*Myd88*^−/−^ mice and 2D2Tg mice after PTX administration. 2D2Tg*Dcir*^−/−^*Myd88*^−/−^ mice (*n* = 10) and 2D2Tg mice (*n* = 8) were twice administered with PTX at day 0 and day 2 and monitored for 30 days. Statistical significance was evaluated using the Mann-Whitney U test (∗, *p* < 0.05). (D) Incidence of EAE-like encephalomyelitis after PTX administration in 2D2Tg*Dcir*^−/−^*Myd88*^−/−^ mice (red) and 2D2Tg mice (sky blue). (E) Histology of spinal cords in 2D2Tg*Dcir*^−/−^*Myd88*^−/−^ mice and 2D2Tg mice with PTX-induced EAE-like encephalomyelitis. The sections of the spinal cords at 30 days after PTX injection were double-stained with H&E and LFB. Representative images are shown (scale bar, 250 μm in low-power fields and 50 μm in high-power fields). Arrows indicate infiltration of inflammatory cells into the spinal cords and arrowheads show demyelination of white matter. (F and G) Inflammation and demyelination scores were assessed with histological examination of the H&E and LFB staining (three spinal cord sections per mouse were evaluated). Each symbol represents an individual mouse, and the bars show the mean ± SEM. Statistical significance was evaluated using the Mann-Whitney U test (∗, *p* < 0.05).

DCIR is an inhibitory type of C-type lectin receptor with an ITIM motif, but the signaling mechanisms underlying DCIR-mediated regulation of immune responses remain unclear. However, the cross-link of human DCIR with anti-human DCIR antibody suppressed cytokine production from plasmacytoid DCs stimulated by a TLR9 agonist or monocyte-derived DCs by a TLR7 and TLR8 agonist, suggesting that DCIR could modulate MyD88 associated with these TLRs.^17^^,^^18^ To evaluate the role of MyD88 in the spontaneous development of EAE-like encephalomyelitis of 2D2Tg*Dcir*^−/−^ mice, we crossed 2D2Tg*Dcir*^−/−^ mice with *Myd88*^−/−^ mice (written as 2D2Tg*Dcir*^−/−^*Myd88*^−/−^ mice) and monitored EAE-like encephalomyelitis under the SPF condition. The incidence of EAE and mortality in 2D2Tg*Dcir*^−/−^*Myd88*^−/−^mice significantly decreased to 7.9% (5/63) and 4.8% (3/63), respectively (Table 3; Figure 3A). The disease severity of spontaneous EAE in 2D2Tg*Dcir*^−/−^*Myd88*^−/−^mice was comparable with that of 2D2Tg mice (Figure 3B), and the maximal clinical score of 2D2Tg*Dcir*^−/−^*Myd88*^−/−^mice also decreased to a similar extent as 2D2Tg mice (2.0 ± 0.5, Table 3). To further examine the role of MyD88 in the development of EAE-like encephalomyelitis, we administered PTX into 2D2Tg*Dcir*^−/−^*Myd88*^−/−^ mice and 2D2Tg mice. The PTX administration resulted in a lower mean clinical score in 2D2Tg*Dcir*^−/−^*Myd88*^−/−^mice (0.5 ± 0) than in 2D2Tg mice (0.79 ± 0.27), and disease onset in 2D2Tg*Dcir*^−/−^*Myd88*^−/−^mice (10.1 ± 4.5) was significantly delayed compared to that in 2D2Tg mice (18.6 ± 3.0, Figure 3C). Incidence in 2D2Tg*Dcir*^−/−^*Myd88*^−/−^ mice decreased relative to 2D2Tg mice (30% vs. 75%) (Figure 3D; Table 4). H&E and LFB staining showed a lower inflammatory score and demyelination score than 2D2Tg*Dcir*^−/−^ mice (Figures 3E–3G), indicating that MyD88-mediated signaling is responsible for developing EAE-like encephalomyelitis in 2D2Tg*Dcir*^−/−^ mice, and DCIR-mediated signaling potentially regulates TLRs associating with MyD88.

**Table 3:** Spontaneous development of EAE-like symptoms in 2D2Tg*Dcir*^−/−^*Myd88*^−/−^ mice

Strain	incidence	mortality	maximum clinical score	weeks of onset
2D2Tg*Dcir*^−/−^*Myd88*^−/−^	5/63(7.9%)	3/63(4.8%)	2.0 ± 0.5 (affected mice)	16.0 ± 13.0
2D2Tg	2/58(3.4%)	0/58(0%)	2.0 ± 0.71 (affected mice)	12.5 ± 2.12

Data are presented as means ± SD.

**Table 4:** EAE-like encephalomyelitis in 2D2Tg*Dcir*^−/−^*Myd88*^−/−^ mice after PTX administration

Strain	incidence	mortality	maximum clinical score	days of onset
2D2Tg*Dcir*^−/−^*Myd88*^−/−^	3/10(30%)	0/10(0%)	0.79 ± 0.27 (affected mice)	10.1 ± 4.5
2D2Tg	6/8(75%)	0/8(0%)	0.5 ± 0 (affected mice)	18.6 ± 3.0^a^

Data are presented as means ± SD.

a *p* < 0.05, two-tailed unpaired Student’s *t* test.

### DCIR-mediated signaling downregulates TLR7 signaling

**Figure 4:**
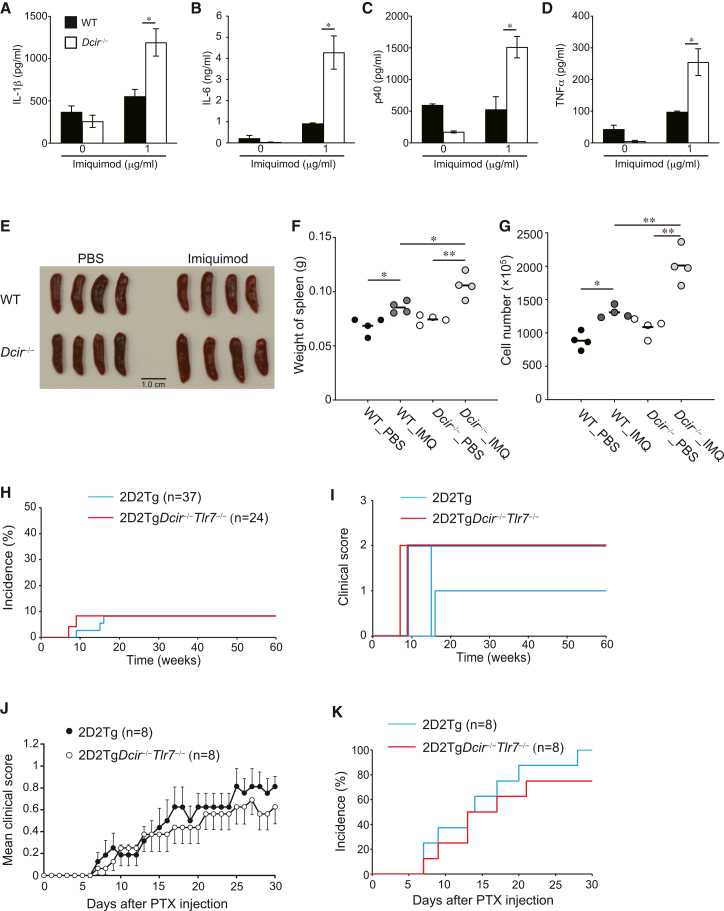
DCIR suppresses TLR7-mediated signaling (A–D) Cytokine production from GMDCs upon stimulation with imiquimod, a TLR7 agonist. GMDCs were stimulated with imiquimod (1 μg/mL) for 3 days, and the amount of cytokine was determined using an ELISA assay. The concentration of cytokine was determined with ELISA (A; IL-1β, B; IL-6, C; p40, D; TNF-α). The bars show mean ± SD of triplicate wells and the data are representative of two independent experiments. ∗∗, *p* < 0.01, ∗, *p* < 0.05. (E) Spleen images of WT and *Dcir*^−/−^ mice after i.v. injection of IMQ. Spleens were removed 24 h after IMQ i.v. injection. (F and G) Spleen weight (F) and splenocyte cell numbers (G). PBS-injected WT mice; solid circles, IMW-injected WT mice; dark gray circles, PBS-injected *Dcir*^−/−^ mice; open circles, IMQ-injected *Dcir*^−/−^ mice; light gray circles. Each symbol shows an individual mouse and a horizontal bar represents the mean for each score. Statistical significances were evaluated using one-way ANOVA with Tukey’s post hoc test (∗∗, *p* < 0.01, ∗, *p* < 0.05). The data are representative of two independent experiments (E–G). (H) Spontaneous EAE-like encephalomyelitis symptoms in 2D2Tg*Dcir*^−/−^*Tlr7*^−/−^ mice. 2D2Tg mice (*n* = 37) and 2D2Tg*Dcir*^−/−^*Tlr7*^−/−^ mice (*n* = 24) were monitored for 60 weeks 2D2Tg mice (sky blue) and 2D2Tg*Dcir*^−/−^*Tlr7*^−/−^ mice (red). (I) Disease severity of spontaneous development of EAE-like encephalomyelitis symptoms in 2D2Tg mice (sky blue) and 2D2Tg*Dcir*^−/−^*Tlr7*^−/−^ mice (red). (J) PTX administration into 2D2Tg mice and 2D2Tg*Dcir*^−/−^*Tlr7*^−/−^ mice. 2D2Tg mice (*n* = 10) and 2D2Tg*Dcir*^−/−^*Tlr7*^−/−^ mice (*n* = 8) were administered pTx twice, at day 0 and day 2, and monitored for 30 days. Statistical significance was evaluated using the Mann-Whitney U test. (K) Incidence of EAE-like encephalomyelitis after PTX administration in 2D2Tg mice (sky blue) and 2D2Tg*Dcir*^−/−^*Tlr7*^−/−^ mice (red). The data were representative of two independent experiments.

Our previous study revealed that *Dcir*^−/−^ DCs upregulate their antigen-presenting ability to T cells and increase gene expression related to receptor signaling, such as *Tlr7*.^6^ Our data in this study strongly support the notion that murine DCIR can downregulate the responses of TLRs, which are associated with MyD88. We differentiated DCs from bone marrow (BM) cells with GM-CSF (GM-DCs) and stimulated GM-DCs with TLR3 (Poly(I:C)), TLR4 (LPS), TLR7 (IMQ), and TLR9 (ODN1668) agonists. TLR3 is known to associate with TRIF, an additional signal adapter protein in the TLR family. *Dcir*^−/−^ GM-DCs remarkably increased IL-1β, IL-6, p40, and TNF-α cytokine production in response to IMQ, a TLR7 agonist, compared to WT GM-DCs. At the same time, DCIR deficiency in DCs had no effect on cytokine production in response to TLR4 and TLR9 agonists (Figures 4A–4D; Figures S2A–S2D). Moreover, *Dcir*^−/−^ DCs didn’t increase cytokine production upon stimulation of Poly(I:C), a TLR3 agonist (Figures S2A–S2D). To further examine the DCIR-mediated regulation of TLR7, we injected IMQ into WT and 2D2Tg*Dcir*^−/−^ mice, followed by an evaluation of spleen weight and the number of splenocytes. IMQ injection increased spleen weight and the number of splenocytes in WT mice, and DCIR deficiency showed higher spleen weight and cell number than those of WT mice (Figures 4E–4G), suggesting the involvement of DCIR in the TLR7-mediated signal. Furthermore, we applied IMQ-induced psoriasis in 2D2Tg and 2D2Tg*Dcir*^−/−^ mice. The psoriasiform skin inflammation was enhanced in 2D2Tg*Dcir*^−/−^ mice than in 2D2Tg mice (Figures S3A and S3B). DCIR deficiency significantly increased the severity score of redness and scale to that of WT mice (Figures S3C–S3E). Increased thickness of the epidermis in 2D2Tg*Dcir*^−/−^ mice was observed in ear sections by H&E staining (Figures S3F and S3G). We detected the expression level of TLR7 in GM-DCs and found that the expression of TLR7 in GM-DCs was comparable between WT and *Dcir*^−/−^ GM-DCs (Figures S4A and S4B). Furthermore, the expression of TLR7 in splenic CD11c+ cells and CD11b+ cells, as well as in bone marrow CD11c+ cells and CD11b+ cells, was comparable between WT and *Dcir*^−/−^ mice (Figures S4C–S4F).

To confirm the DCIR-mediated regulation of TLR7 signaling, we generated 2D2Tg*Dcir*^−/−^*Tlr7*^−/−^ mice by crossing 2D2*Dcir*^−/−^mice with *Tlr7*^−/−^ mice. We monitored the spontaneous development of EAE-like encephalomyelitis under the SPF condition, and the incidence of EAE and mortality in 2D2Tg*Dcir*^−/−^*Tlr7*^−/−^ mice significantly decreased to 8.3% (2/24) and 4.2% (1/24), respectively (Table 5; Figure 4H). The disease severity of EAE-like encephalomyelitis in 2D2Tg*Dcir*^−/−^*Tlr7*^−/−^ mice was comparable to that of 2D2Tg mice (Figure 4I). As PTX administration induced severe EAE-like encephalomyelitis in 2D2Tg*Dcir*^−/−^ mice, we examined the effect of PTX administration on clinical score in 2D2Tg*Dcir*^−/−^*Tlr7*^−/−^ mice and 2D2Tg mice. The PTX administration into 2D2Tg*Dcir*^−/−^*Tlr7*^−/−^ mice caused incidence and clinical scores similar to those in 2D2Tg mice, indicating that TLR7 is responsible for the spontaneous development of EAE-like encephalomyelitis in 2D2Tg*Dcir*^−/−^ mice (Figures 4J and 4K; Table 6), suggesting that DCIR-mediated signaling contributes to downregulation of TLR7 responses which are associated with MyD88.

**Table 5:** Spontaneous development of EAE-like symptoms in 2D2Tg*Dcir*^−/−^*Tlr7*^−/−^ mice

Strain	incidence	mortality	maximum clinical score	days of onset
2D2Tg*Dcir*^−/−^*Tlr*7^−/−^	2/24(8.3%)	1/24(4.2%)	2.0 ± 0.0 (affected mice)	45.5 ± 24.7
2D2Tg	3/37(8.1%)	0/37(0%)	1.7 ± 0.58 (affected mice)	93.3 ± 26.5

Data are presented as means ± SD.

**Table 6:** EAE-like encephalomyelitis in 2D2Tg*Dcir*^−/−^*Tlr7*^−/−^ mice after PTX administration

Strain	incidence	mortality	maximum clinical score	days of onset
2D2Tg*Dcir*^−/−^*Tlr*7^−/−^	6/8(75%)	0/8(0%)	0.75 ± 0.27 (affected mice)	14.5 ± 5.3
2D2Tg	8/8(100%)	0/8(0%)	0.94 ± 0.32 (affected mice)	11.5 ± 4.9

Data are presented as means ± SD.

### U11snRNA, an endogenous ligand for TLR7, is detectable in 2D2Tg*Dcir*^−/−^ mice

**Figure 5:**
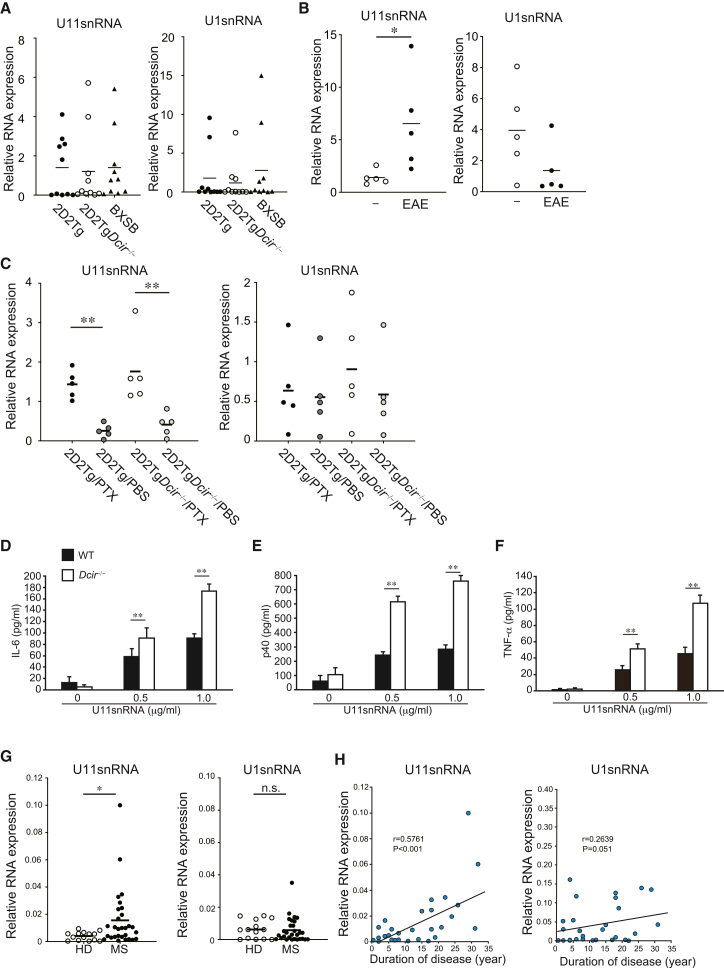
An endogenous ligand of TLR7, U11snRNA, was detected in 2D2Tg, 2D2Tg*Dcir*^−/−^ mice, and MS patients (A) Serum U11snRNA in 2D2Tg mice and 2D2Tg*Dcir*^−/−^ mice. Sera were prepared from 2D2Tg mice, 2D2Tg*Dcir*^−/−^ mice, and BXSB/MpJ-*Yaa* mice, and U11snRNA was determined with a PCR method. Statistical significances are evaluated by one-way ANOVA with Dunnett’s post hoc test. (B) Serum U11snRNA after immunization with MOG_35-55_ peptide. WT mice (*n* = 5) were immunized with MOG_35-55_ peptide and were intraperitoneally administered with PTX at days 0 and 2. Serums were collected 7 days after immunization. Sera from non-treated WT mice (*n* = 5) were used as controls. Each symbol represents an individual mouse, and a horizontal bar presents the mean of each score. ∗, *p* < 0.05. (C) Serum level of snRNA after PTX injection. Sera at 7 days after twice PTX injection were collected from 2D2Tg mice (*n* = 5) and 2D2Tg*Dcir*^−/−^ mice (*n* = 5), and snRNA was determined using a PCR method. Statistical significances are evaluated by one-way ANOVA with Tukey’s post hoc test. (D–F) Cytokine production from splenocytes upon stimulation with U11snRNA. Splenocytes were stimulated with U11snRNA (0.5 and 1.0 μg/mL) for 3 days, and the cytokines were determined with ELISA (D; IL-6, E; p40, F; TNF-α). The bars show mean ± SD of triplicate wells and the data are representative of two independent experiments (C–E). ∗∗, *p* < 0.01. (G) qPCR detection of the indicated genes in the serum from multiple sclerosis (solid circle) and healthy donors (HD) (open circle). Each symbol represents an individual patient, and a horizontal bar represents the mean of each score. ∗, *p* < 0.05. (H) Correlation analysis between the relative amount of the indicated RNA and the duration of MS disease. The Pearson correlation coefficient and its associated *p* value were calculated

To examine the mechanisms that lead to the induction of the spontaneous development of EAE-like encephalomyelitis in 2D2Tg*Dcir*^−/−^ mice, we focused on endogenous components that could stimulate immune responses. TLR7, which is associated with autoimmune diseases, is an innate immune receptor that detects viral single-strand RNA (ssRNA). However, U1 small nuclear RNA and U11 small nuclear RNA (U11snRNA) are also known as endogenous ligands for TLR7.^19^^,^^20^ U11snRNA robustly activates TLR7 signaling, as observed in BXSB/MpJ-*Yaa* mice, a mouse disease model for SLE,^21^ and an elevated relative amount of serum U11snRNA was observed with age. SLE and (RA) patients also have high serum levels of U11snRNA, indicating that U11snRNA is an endogenous pathogenic ligand of TLR7 to develop autoimmune diseases.^19^ Thus, we hypothesized that serum U11snRNA is detectable in 2D2Tg*Dcir*^−/−^ mice. We found that, similarly to BXSB/MpJ-*Yaa* mice, the serum U11snRNAs were detected in 2D2Tg and 2D2Tg*Dcir*^−/−^ mice, although there was no significant difference between them (Figure 5A). EAE-induced WT mice, which were immunized by MOG_35-55_ peptide and administered by PTX, increased the relative amount of serum U11snRNA, but not U1snRNA, compared to normal WT mice (Figure 5B), indicating that U11snRNA is a potential pathogenic factor in EAE. Moreover, PTX administration raised U11snRNA in sera, but not U1snRNA, in 2D2Tg mice and 2D2Tg*Dcir*^−/−^ mice (Figure 5C). As we detected IL-6, a pro-inflammatory cytokine, in the serum of 2D2Tg*Dcir*^−/−^ mice, PTX induced an inflammatory response (Figure S5).^22^ Therefore, these data demonstrate that U11snRNA is detectable even under normal conditions and is increased in serum under inflammatory conditions.

To understand the molecular basis of the U11snRNA-induced responses, cytokine production of p40, IL-6, and TNF-α from WT and *Dcir*^−/−^ splenocytes was determined after stimulation with U11snRNA. U11snRNA, which was complexed with cationic liposome, induced p40, IL-6, and TNF-α pro-inflammatory cytokines more strongly in *Dcir*^−/−^ splenocytes than in WT splenocytes (Figures 5D–5F). We further examined the relative serum amount of U11snRNA in MS patients, as Negishi et al. had detected U11snRNA in RA and SLE patients. A higher relative serum amount of U11snRNA was detectable in MS patients than in healthy controls (HD) (Figure 5G). We found a significant correlation (r = 0.5761, *p* < 0.01) between serum U11snRNA and the duration of MS symptoms in MS patients, but not between U1snRNA and the duration of MS symptoms (r = 0.2639) (Figure 5H). These data suggest that DCIR deficiency increases the response to self-derived nucleic acids, an endogenous ligand for TLR7, as well as a pathogenic factor in autoimmune diseases, and the persistent activation of TLR7 by U11snRNA is responsible for driving spontaneous autoimmunity in the CNS.

## Discussion

Autoreactive T cells are necessary for the development of autoimmune encephalomyelitis, but the presence of autoreactive T cells alone is insufficient to cause the disease spontaneously in 2D2Tg mice.^16^ In the present study, we have demonstrated that DCIR, an inhibitory CLR that recognizes an endogenous ligand, is a regulatory factor that suppresses the autoreactivity of immune responses in the CNS. DCIR deficiency in 2D2Tg mice enhanced the development of EAE-like encephalomyelitis, and MyD88-dependent signaling is responsible for the development of EAE-like encephalomyelitis in 2D2*Dcir*^−/−^ mice. Our findings revealed that U11snRNA, an endogenous RNA ligand for TLR7, is a pathogenic factor that exacerbates EAE conditions via the TLR7 pathway, explaining the puzzling roles of TLR7 and DCIR in the development of autoimmune diseases.

DCIR is expressed on DCs, which present antigens to T cells and dictate the differentiation of naive T cells to effector T cells. *Dcir*^−/−^ mice spontaneously developed autoimmune-like diseases, like enthesitis and sialadenitis, and showed more severe EAE than WT mice.^8^^,^^10^ The deficiency of DCIR increased gene expression associated with antigen-presenting ability.^6^ Therefore, these studies provide a rationale that DCIR is a sufficient condition for the pathogenesis of EAE-like encephalomyelitis in 2D2Tg mice. Indeed, DCIR deficiency in 2D2Tg mice increased EAE-like encephalomyelitis and T cells from 2D2Tg*Dcir*^−/−^ mice enhanced cytokine production and proliferation in response to MOG_35-55_ peptide. Splenic CD11c^hi^ DCs isolated from *Dcir*^−/−^ mice promoted IFN-γ production in T cells more than WT DCs in the presence of soluble anti-CD3 antibody.^9^ Moreover, neuraminidase treatment, which increases the expression of endogenous DCIR ligands, reduced cytokine production from 2D2 T cells in a DCIR-dependent manner.^6^ Thus, uncontrollable DC activation resulting from the loss of DCIR-mediated regulation stimulates autoreactive T cells.

The aggravation of EAE symptoms in PTX-injected 2D2Tg*Dcir*^−/−^ mice demonstrates that inflammation, defective DCIR, and autoreactive TCR are essential pathogenic factors in autoimmune disease. In EAE, PTX is used to promote the disease symptoms. PTX can disrupt the blood-brain barrier (BBB) and increase its permeability, allowing immune cells to invade the brain.^23^ In addition, PTX-stimulated splenic cells secreted pro-inflammatory cytokines, such as TNF-α and IL-6, likely through TLR4-dependent signaling pathways.^24^^,^^25^^,^^26^ PTX also induces pro-inflammatory cytokines through the DAP12, FcRγ, and MyD88 signal adapter proteins.^27^ A deficiency of DCIR upregulates the antigen presentation ability of DCs, resulting in enhanced T cell responses.^6^ Thus, PTX injection and defective DCIR synergistically induce aberrant inflammatory responses and enhanced antigen presentation, which could trigger uncontrolled immune reactions. On the other hand, T cell activation requires TCR-mediated antigen stimulation; however, bystander T cell activation is non-specifically initiated in a TCR-independent manner through pro-inflammatory cytokines, and the bystander-activated T cells contribute to autoimmune neuroinflammation.^28^^,^^29^ Although antigen-specific Th1 and Th17 cells are responsible for autoimmune neuroinflammation, the infiltration of non-myelin-specific CD4^+^ T cells into the spinal cord is attributed to EAE development.^30^^,^^31^^,^^32^ Therefore, DCIR deficiency exacerbates PTX-induced cytokine milieus by excessive stimulation of myeloid cells, which activate myelin-specific CD4^+^ T cells in a TCR-independent manner, indicating the importance of antigen specificity and chronic inflammation in autoimmune diseases.

DCIR functions as an inhibitory signaling receptor in immune regulation. DCIR was defined as a receptor that downregulates immune responses through TLR pathways.^18^^,^^33^ Meyer-Wentrup et al. also showed that DCIR is translocated into the endosomal/lysosomal compartments in a clathrin-dependent manner. TLR7 is located in endosomes and recruits MyD88 at the cytoplasmic site, and the ligand-binding site of TLR7 is in the lumen of endosomes.^34^ These findings indicate that DCIR and TLR7 are likely co-localized in endosomal compartments; however, the mechanism by which DCIR downregulates TLR7-mediated signaling remains unresolved. Interestingly, an ITIM-harboring immunoglobulin-like receptor, gp49B, negatively regulates osteoclast formation by inducing the association of SHP-1 with TRAF6.^35^ DCIR-activated SHP-1 may form a complex with TRAF6, an adapter protein that associates with MyD88,^36^ and thereby downregulate MyD88-induced signaling. However, the exact substrate of SHP-1 binding to DCIR in MyD88-induced signaling must be elucidated.

U11snRNA is a self-derived RNA involved in the development of autoimmunity.^19^ U11snRNA is a component of the protein assembly for the minor-class splicing pathways and is initially located in the cell nucleus. Our study, as well as another, detected U11snRNA and other endogenous RNAs in sera. The non-specific release of U11snRNA activates the TLR7-Myd88 axis, resulting in the development and exacerbation of EAE and MS, and DCIR regulates and inhibits the TLR7-induced neuroinflammation. However, the sources of U11snRNA in sera, which are located in the cytoplasm and the cell nucleus, remain unclear. In our study, U11snRNA increases in serum after EAE induction and PTX *in vivo* injection; therefore, it may be released from damaged or dying cells as damage-associated molecular patterns (DAMPs).^12^ As Epstein-Barr virus (EBV) is associated with MS, the viral infection might trigger non-specific U11snRNA release through immune responses and neuroinflammation.^37^ Inflammation promotes the release of microvesicles (MVs)/exosomes from microglia/macrophages and epithelial cells. MVs/exosomes contain various constituents of nucleic acids (mRNA, microRNA, and DNA), proteins, and lipids and function as mediators of intercellular communication.^38^^,^^39^^,^^40^^,^^41^ The concentration of MVs from myeloid cells in MS patients and mice with established EAE increased during neuroinflammation.^38^ Although MVs/exosomes are relatively stable, their membrane integrity could be compromised by physical stress or biological factors, such as proteases and complement factors, potentially leading to the release of their contents into the serum.

Intraperitoneal injection of PTX upregulates U11snRNA in serum. However, the mechanisms of U11snRNA release by PTX injection remain elusive. PTX elicits inflammation via DAP12-, FcRγ-, and MyD88-dependent signaling pathways.^27^ As we detected serum IL-6 at 6 and 24 h after intraperitoneal PTX injection (Figure S5), the PTX-induced inflammation might initiate U11snRNA release. The other possibility is that PTX releases U11snRNA via potential or unidentified receptors. Coated PTX had binding capacity to C-type lectin receptors, such as DCSIGN, SIGNR1, SIGNR2, and MGL-1.^27^ Although it is not yet clear whether these receptors activate or inhibit immune responses, PTX might trigger U11snRNA release by binding to these immunoreceptors or unknown receptors. Further studies are, however, required to clarify how U11snRNA is released into the sera during the development of EAE and MS.

MyD88 plays a broader role in EAE pathogenesis. MyD88-deficient mice were not susceptible to EAE, whereas TLR9-knockout mice, which recruit MyD88 as an adapter protein, were partially susceptible.^23^ Moreover, IL-1RI deficiency, which binds IL-1β and recruits MyD88, ameliorated EAE.^42^ Our data showed that 2D2Tg*Dcir*^−/−^*Myd88*^−/−^ mice showed less and almost no EAE-like encephalomyelitis than 2D2Tg mice. Therefore, it is reasonable to assume that 2D2Tg*Myd88*^−/−^ mice are resistant to EAE, independent of DCIR deficiency, at a similar level to 2D2Tg*Dcir*^−/−^*Myd88*^−/−^ mice. Chimeric mice with WT BM reconstitution in *Myd88*^−/−^ mice exhibited a delay in disease onset and a reduction of severity in EAE, compared to chimeric mice with WT BM in WT mice,^12^ demonstrating that MyD88 expression in non-hematopoietic cells is vital for disease development. In contrast, chimeric mice with the donor *Myd88*^−/−^ BM into WT mice were completely protected from EAE. They did not produce T cell recall responses to MOG, indicating that MyD88 expression in hematopoietic cells is important for priming, proliferating, and activating autoreactive T cells and indispensable for complete disease onset. Because DCIR mainly signals through myeloid cells, such as DCs and macrophages/monocytes, but not non-hematopoietic cells, it is plausible that DCIR inhibits MyD88-mediated signaling in hematopoietic cells, which are crucial for autoreactive T cell activation, and is one of the key inhibitory receptors that prevent the induction of EAE and MS.

Murine DCIR deficiency exacerbated the clinical score of EAE, and neuraminidase injection into WT mice with established EAE ameliorated EAE symptoms.^10^^,^^11^ However, the association of human DCIR functions with MS severity remains unknown. The International Multiple Sclerosis Genetics Consortium (IMSGC) reported genetic variants associated with MS severity, collecting data from North America, Europe, and Australia.^2^ The Genome-Wide Association Study (GWAS) identified 29 genetic variants in the human CLEC4A locus on chromosome 12, but there was no statistically significant association with MS progression. rs2024301 and rs117213717 single-nucleotide variants (SNVs) localize at exon 2 of the human CLEC4A gene encoding amino acids in the cytoplasmic region. rs2024301 SNV changes histidine into leucine, and rs117213717 SNV changes glycine into arginine. Interestingly, histidine and glycine are conserved in CLEC4A proteins across various species, indicating that the identified variants may influence the disease outcome (Figure S6). However, the structural significance of MS variants in human DCIR should be further investigated. In addition, although the report from IMSGC is one of the most extensive genetic studies, a GWAS in Asian MS patients would provide insight into the relationship between human DCIR and the severity of MS.

Defective DCIR spontaneously develops an autoimmune central nervous system disease in genetically modified mice with myelin-specific TCR. DCIR is involved in the downregulation of the TLR7-Myd88 axis, which is activated by endogenous ligand U11snRNA. Pro-inflammatory cytokines released from myeloid cells activate autoreactive T cells, even in the absence of antigen-specific TCR stimulation. Therefore, DCIR is a crucial negative regulator of EAE and MS induction. However, the role of NA2, an endogenous ligand for DCIR, in regulating TLR7 through DCIR remains to be elucidated. Further studies dissecting the molecular mechanisms that induce DCIR activation will provide a deeper understanding of the pathological mechanisms underlying central autoinflammation, suggesting alternative therapeutics targeting DCIR and TLR7 for the treatment of immune-related diseases.

### Limitations of the study

This study has several limitations. First, many *in vivo* experiments were conducting suing animal disease models of gene-modified mice. Unlike experimentally induced disease models, 2D2Tg mice with DCIR deficiency spontaneously developed EAE-like encephalomyelitis, but 2D2Tg mice had only autoreactive T cells expressing Vα3.2 and Vβ11. This does not fully recapitulate the complexity of human neuroinflammation. Although human samples of MS patients were analyzed, it should be noted whether these findings extrapolate to human autoimmune diseases. Second, the study also relied on the EAE model, which has been accepted as a standard animal model for MS. However, it does not fully recapitulate the pathogenesis of human multiple sclerosis, particularly its progression. MS can be categorized into several clinical subtypes based on clinical progression, but EAE causes chronic neuroinflammation after disease induction. Moreover, in EAE, lesions occur in the spinal cord rather than the brain, although in humans they are primarily located in the brain. Third, the precise molecular mechanisms by which DCIR-mediated signaling downregulates dendritic cell functions remain to be fully elucidated. In particular, the downstream signaling pathways to regulate the TLR7-MyD88 signal and the release ways of an endogenous ligand for TLR7 remain to be clarified. DCIR has a docking site for tyrosine phosphatases, such as SHP-1, but the direct substrate of MyD88 signaling pathways, which depend on serine-threonine kinases, remains unidentified. Biochemical analyses using a DCIR-overexpressing cell line would provide more evidence for signal regulation downstream of DCIR. In addition, because we detected serum U11snRNA, an endogenous ligand for TLR7, after EAE and PTX administration, inflammation likely initiates U11snRNA release into the serum, but the precise mechanism of U11snRNA release from the intracellular compartment was not analyzed in this study. Finally, the human samples were from a single-center study with a relatively small sample size, which may limit the generalizability of the relationship between U11snRNA expression and disease duration. It is difficult to eliminate the potential effects of therapeutic interventions. Despite these limitations, our findings provide valuable insights into DCIR-mediated inhibitory regulation of autoreactive T cells that cause autoimmune diseases.

## Resource availability

### Lead contact

Requests for further information and resources should be directed to and will be fulfilled by the lead contact, Tomonori Kaifu (kaifutom@iwate-med.ac.jp).

### Materials availability

This study did not generate new unique reagents.

### Data and code availability

The GWAS data of multiple sclerosis used in this study are available from the International Multiple Sclerosis Genetics Consortium (IMSGC). This study did not create new code. No other resources have been created in this study. Source data of other figures will be provided upon request.

## Acknowledgments

We thank A. Akitsu, Y. Matsuoka, and A. Tobinai-Sugawara for their technical support and helpful discussions. This work was supported in part by a grant for Grant-in-Aid for Scientific Research (C) (grant number, 23500489) of 10.13039/501100001691Japan Society for the Promotion of Science to T.K. and the 10.13039/100008732Uehara Memorial Foundation.

## Author contributions

A.F., S.N., D.U., and T.K. conducted most of the experiments; A.F., S.N., and Y.C. conducted flow cytometer and histological analysis; T.K. performed data analysis of flow cytometer; T.K. prepared cellular samples; K.S. produced 2D2Tg*Dcir*^−/−^ mice by crossing 2D2Tg mice and *Dcir*^−/−^ mice; A.F. and T.K. wrote the draft manuscript, and T.K. edited the draft manuscript. Y.I., Y.M., and A.N. supervised the project, and T.K. organized this project. All authors discussed the data and approved the final manuscript.

## Declaration of interests

The authors declare that they have no conflict of interest.

## Declaration of generative AI and AI-assisted technologies in the writing process

The authors declare that no AI was used to generate or evaluate the data. To improve readability and language, the authors used AI-assisted proofreading software (Grammarly) but reviewed the content themselves and take full responsibility for the article.

## STAR★Methods

### Key resources table

**Table:** 

REAGENT or RESOURCE	SOURCE	IDENTIFIER
**Antibodies**		

Anti mouse CD3 PE/eFluor610(Clone17A2)	eBioscence	Cat#61-0032-82;RRID;AB_2815286
Anti mouse CD4 pacific blue(CloneRM4-5)	BioLegend	Cat#100531;RRID;AB_493374
Anti mouse CD8 AlexaFluor700(Clone53-6.7)	BioLegend	Cat#100729;RRID;AB_493702
Anti mouse Vα3.2 FITC(CloneRR3-16)	BioLegend	Cat#135403;RRID;AB_1937236
Anti mouse Vβ11 PE(CloneRR3-15)	BioLegend	Cat#10613472;RRID;AB_10613472
Anti mouse CD3 PE/Cy5(Clone145-2C11)	BioLegend	Cat#100310;RRID;AB_312675
Anti mouse CD4 FITC(CloneGK1.5)	BD Bioscience	Cat#553729;RRID;AB_395013
Anti mouse TNFαPE(CloneMP6-XT22)	BioLegend	Cat#506306;RRID;AB_315426
Anti mouse IL-17 PE/Cy7(CloneTC11-18H10.1)	BioLegend	Cat#506922;RRID;AB_2616697
Anti mouse IFN-γ APC/Cy7(CloneXMG1.2)	BioLegend	Cat#505849;RRID;AB_10613472
Anti mouse Ki67 APC(Clone16A8)	BioLegend	Cat#652405;RRID;AB_2561929
Anti moouse TLR7 PE(CloneA94B10)	BioLegend	Cat#160003;RRID;AB_2860749
Anti mouse CD11b FITC(CloneM1/70)	BioLegend	Cat#1010206;RRID;AB_312789
Anti mouse CD11c PE/Cy7(CloneN418)	BioLegend	Cat#117217;RRID;AB_493569
Anti mouse CD11b APC/Cy7(CloneM1/70)	BioLegend	Cat#101226;RRID;AB_830641
Anti mouse CD11c FITC(CloneN418)	BioLegend	Cat#117305;RRID;AB_313774
Anti mouse B220 AlexaFluor700(CloneRA3-6B2)	BioLegend	Cat#103231;RRID;AB_493716
Anti mouse CD4 PE/Cy7(CloneRM4-5)	BioLegend	Cat#100527;RRID;AB_312728
Anti mouse CD8 APC(Clone53-6.7)	BioLegend	Cat#100711;RRID;AB_312750

**Biological samples**		

Patient-derived sera	Tohoku Medical and Pharmaceutical University	https://www.hosp.tohoku-mpu.ac.jp
Collagenase VIII(Clostridium histolyticum)	Sigma-Aldrich	C2139

**Chemicals, peptides, and recombinant proteins**		

Pertussis toxin	List labs	#180
Human GM-CSF	Peprotech	AF-300-03
MOG35-55 (MEVGWYRSPFSRVVHLYRNGK)	KITAYAMA LABES CO., LTD	N/A
Imiquimod	InvivoGen	#99011-78-6
Beselna Cream (5% Imiquimod)	Mochida Pharmaceutical	224130002
Poly(I:C)	InvivoGen	31852-29-6
LPS	Sigma-Aldrich	0127∗B8(L3129)
ODN1669	Fasmac Co., Ltd.	N/A

**Critical commercial assays**		

ELISA Ready-Set-Go!(IL-17)	Affymetrix eBioscience	88-7371
ELISA MAX Standard Set (IFN-γ)	BioLegend	430801
ELISA MAX Standard Set (IL-6)	BioLegend	431303
ELISA MAX Standard Set (TNF-α)	BioLegend	430903
BD OptEIA (p40)	BD Biosciences	555165
IL-1β ELISA kit	ThermoFisher	88-7013A-88
NucleoSpin miRNA Plasma	TAKARA bio	740981.10
PrimeScrip RT reagent kit with gDNA Eraser	TAKARA bio	RR047A

**Experimental models: Organisms/strains**		

Mouse: C57BL/6-Tg(Tcra2D2,Tcrb2D2)1Kuch/J	The Jackson Laboratory	#006912
Mouse:BXSB/MpJ-Yaa	Japan SLC, Inc.	#010601
Mouse:C57BL/6J	Japan SLC, Inc.	N/A

**Oligonucleotides**		

Primer for mouse Rnu11, see Table S1	ThermoFisher	N/A
Primer for human Rnu11, see Table S1	ThermoFisher	N/A
Primer for mouse Rnu1, see Table S1	ThermoFisher	N/A
Primer for EGFP, see Table S1	ThermoFisher	N/A
Murine U11snRNA sequence, see Table S2	Fasmac Co., Ltd.	N/A
EGFP mRNA, see Table S2	Fasmac Co., Ltd.	N/A

**Software and algorithms**		

NanoZoomer-SQ	Hamamatsu Photonics	N/A
GraphPad Prism v9.0	GraphPad Software	https://www.graphpad.com
FlowJo	FlowJo, LLC	https://www.flowjo.com
Clustal W	EMBL-EBI	https://www.genome.jp/tools-bin/clustalw

### Experimental model and study participant details

#### Animals

*Dcir*^−/−^ mice were generated as described and were backcrossed to C57BL/6J at 12 generations. 2D2 TCR transgenic (2D2Tg) mice were purchased from the Jackson Laboratory (#006912). To generate 2D2Tg*Dcir*^−/−^ mice, *Dcir*^−/−^ mice were crossed into 2D2Tg mice in our mouse facility. 2D2Tg*Dcir*^−/−^*Myd88*^−/−^ mice were generated by crossing 2D2Tg*Dcir*^−/−^ mice with *Myd88*^−/−^ mice. *Tlr7*^−/−^ mice were kindly provided by Dr. Miyake and Dr. Fukui (The University of Tokyo, Japan). 2D2Tg*Dcir*^−/−^*Tlr7*^−/−^ mice were generated by crossing 2D2Tg*Dcir*^−/−^ mice with *Tlr7*^−/−^ mice. All murine strains were born following the expected Mendelian frequency and typically developed with fertility. BXSB/MpJ-*Yaa* mice were kept in our animal facility. All the mice used in these experiments were male and female with 8-12 weeks old, and these age- and sex-matched control C57BL/6J mice were purchased from Japan SLC, Inc. (Shizuoka, Japan). All mice were housed in the animal facilities of Tohoku Medical and Pharmaceutical University (24024-cn, 25029-cn), Iwate Medical University (7-11), or Tokyo University of Science (S15016), and animal experiments were conducted in accordance with the guidelines of each university. The Animal Experiment Committees approved them.

#### Cell culture

Bone marrow-derived dendritic cells: Bone marrow cells were isolated by flushing the BM cavity of the femurs and tibias with R10. Red blood cells were destroyed on ice in a hemolysis buffer for 10 min. The bone marrow cells were cultured at a concentration of 2 × 10^6^ cells in 10 ml R10 in a non-treated 100 mm dish with 20 ng/ml of GM-CSF (Peprotech). Three days after incubation, 10 ml of R10 containing 20 ng/ml of GM-CSF was added to the dish. At 6 and 8 days, 10 ml of supernatant was collected, and the cell pellet, after centrifugation at 1500 rpm for 5 min, was resuspended in an equal volume of R10 containing 20 ng/ml of GM-CSF, followed by the restoration of the suspension into the dish. At 10 days, cells in the supernatant were used as GM-CSF-induced dendritic cells (GM-DCs).

#### Human data

All patients were recruited from the Division of Neurology and the Division of Hematology and Rheumatology at Tohoku Medical and Pharmaceutical University until the target sample size was achieved. MS patients fulfilled the 2017 revision of the McDonald criteria (Thompson et al., 2018), and disease severity was determined according to the Expanded Disability Status Scale (EDSS). 18 were female (the average age is 44.7 years old) and 15 were male (the average age is 41.3 years old). All patients had relapsing-remitting multiple sclerosis and provided informed consent for the study. All but one were receiving disese-modifying therapy. This study conformed to ethical standards of national legislation and was approved by the clinical research ethics board of Tohoku Medical and Pharmaceutical Hospital (approval numbers: 2022-2-026 and 2023-2-054). Healthy donor blood samples were available from the Japan Red Cross Society (authorization numbers: R060037 and R060038).

### Method details

#### Peptides

Mouse MOG_35-55_ (MEVGWYRSPFSRVVHLYRNGK) was synthesized by KITAYAMA LABES CO., LTD (Nagano, Japan).

#### EAE-like encephalomyelitis induction by pertussis toxin injection

2D2Tg, 2D2Tg*Dcir*^−/−^, 2D2Tg*Dcir*^−/−^*Myd88*^−/−^, and 2D2Tg*Dcir*^−/−^*Tlr7*^−/−^ mice were intraperitoneally injected with 200 ng of pertussis toxin in PBS on days 0 and 2. Clinical scores of EAE were monitored for 30 days according to the following grades: 0, no disease; 0.5, partial limp tail; 1, paralyzed tail; 1.5, paralyzed in one hind limb; 2, both hind limbs paralyzed; 2.5, weakness in one forelimb; 3, both forelimbs weakened; 4, forelimbs paralyzed; 5, moribund. The mean clinical score was determined by averaging the scores of all mice in each group. The cumulative score is the sum of the daily clinical scores for individual mice and is reported as the average for each group. The average maximal score is calculated as an average of the maximal clinical score of individual mice in each group.

#### Serum pro-inflammatory cytokine after PTX injection

2D2Tg mice (8 weeks old) were intraperitoneally injected with PTX (200 ng per mouse). Blood samples were obtained from the facial vein at 3 days before injection, and 6 hr after injection and whole blood specimens were collected with cardiac puncture under anesthesia at 24 hr after injection. The sera were stored at –80°C until use.

#### T cell response to MOG_35-55_ peptide

Single cells from the spleens of 2D2Tg mice and 2D2Tg*Dcir*^−/−^ mice were harvested and seeded at 5 × 10^5^ cells per well in 96-well round-bottom plates. The cells were cultured in the presence of 10 μg/ml and 100 μg/ml of MOG_35-55_ peptide for 72 hr in RPMI supplemented with 0.1 mM of β-mercaptoethanol, 100 units/ml of penicillin, 100 μg/ml of streptomycin, and 10% heat-inactivated fetal bovine serum (the culture medium is called R10). These cells were centrifuged at 1500 rpm for 5 min, and the supernatant was stored at -80°C until use.

#### Isolation of infiltrated cells in spinal cords

Mice at 15 days after EAE induction with PTX administration were anesthetized with the anesthetic agents described above and perfused with PBS by gravity-driven perfusion. The spinal columns were entirely removed, and the spinal cord was flushed from the column by PBS with an 18-gauge needle-equipped syringe. The spinal cords were digested in 200 U/mL of collagenase VIII from *Clostridium histolyticum* (Sigma-Aldrich) in HBSS at 37°C for 30 min. The digested spinal cords were resuspended in 30% Percoll (GE Healthcare, IL, USA) and overlaid on a 70% Percoll layer in a 15 ml polypropylene tube, followed by centrifugation at 850 G for 20 min at room temperature without acceleration and braking. Infiltrated cells were collected from the interface between 30% and 70% Percoll layers and analyzed using a flow cytometer.

#### Cytokine ELISA

The cytokine concentration in the culture supernatant or in sera was determined by ELISA, according to the manufacturer’s procedures. The concentration of IL-17 was measured with ELISA Ready-Set-Go! sets (Affymetrix eBioscience) and IFN-γ, IL-6, and TNF-α were determined by mouse ELISA MAX (BioLegend). IL-12 (p40) was assayed with BD OptEIA^TM^ Set (BD, San Diego, CA), and IL-1β was measured with mouse IL-1β uncoated ELISA (ThermoFisher).

#### Flow cytometry

A single-cell suspension was prepared by grinding thymuses, lymph nodes, or spleens with a 5-ml syringe (Telmo) through 70 μm cell strainers (Falcon). The cells were subjected to blood cell lysis on ice using a hemolysis buffer (comprising 140 mM NH_4_Cl and 17 mM Tris-HCl, pH 7.2). The Fc receptors on these cells were blocked with 2.4G2, an Fc receptor blocker, at 4°C for 10 min. These cells were washed twice with a FACS buffer containing 2% fetal bovine serum (FBS) in PBS and incubated with fluorescence-conjugated antibodies for 30 min on ice. Commercially available antibodies used in the experiments were described in Table S3. For intracellular staining, cells were stimulated with 50 μg/ml of PMA (Sigma-Aldrich CO. LCC., St.Louis, MO., USA) and 1 μg/ml of ionomycin (Sigma), and 1000-fold diluted Brefeldin A (eBioscience, San Diego, CA, USA) for 6 hr. The cells were stained at the beginning with surface antigens, CD3, for 30 min on ice. At room temperature, these cells were fixed with 4% paraformaldehyde (Nacalaitesque, Japan) for 30 min. After washing, the cells were permeabilized with Foxp3/Transcription Factor Staining Buffer (eBioscience), according to the manufacturer’s instructions. The intracellular antigens were stained for 30 min on ice with antibodies described in Table S3. Dead cells were stained with BD Horizon^TM^ Flexible Viability Stain 510 (BD Biosciences, NJ, USA). The cells were washed twice with FACS buffer and analyzed with an Attune NxT flow cytometer (Thermo Fisher Scientific, MA, USA). The data were analyzed using FlowJo software (Tree Star, Inc., Ashland, OR, USA).

#### Intravenous imiquimod injection

Imiquimod (20 μg per mouse) was intravenously injected, and spleens were dissected 24 hr after injection. The spleen weight was measured by a scale (METTLER TOLEDO, Tokyo, Japan). The splenocytes were harvested by passing them through cell strainers (Corning, Tokyo, Japan) and counted using a Cellometer (Nexcelom Bioscience, MA, USA).

#### Imiquimod-induced skin inflammation and assessing the severity of skin inflammation

Mice daily received 〜14 mg of Beselna Cream (5% IMQ, Mochida Pharmaceutical, Tokyo, Japan) on both ears for 6 consecutive days. Ear thickness was measured daily at the three fields per ear using a micrometer (OZAKI MFG CO., LTD, Japan). The ear swelling was expressed as a percentage of thickness compared to the ear at day 0. Erythema was measured at two fields of the ear, and scales were evaluated at three areas of the ear. Symptom severity was determined on a scale from 0 to 4; 0, no clinical sign; 1, slight; 2, moderate; 3, marked; 4, very marked (scale 0-4). The cumulative score was the sum of erythema and scaling and was shown as a parameter of the severity of inflammation (0-8).

#### Histology

Mice were anesthetized with a combination of three types of anesthetic agents consisting of 0.3 mg /kg of medetomidine (Kyoritsu Seiyaku corporation, Japan), 4 mg/kg of midazolam (Astellas Pharm Inc., Japan), and 5 mg/kg of butorphanol (Meiji Seika Pharma Co., Ltd, Japan) after at least 4 weeks of spontaneous EAE. Mice with PTX-induced EAE were anesthetized at 30 days after PTX infection. The mice were perfused with PBS to wash blood from the spinal cords through the intracardiac route and continuously with a 10% formalin neutral buffer solution (pH 7.4) by gravity-driven perfusion. Amputated spinal cords were further fixed in 10 % formalin solution for 24 hr. Dissected spinal cords were dehydrated through 70%, 85%, 95%, and 100% alcohol, followed by xylene substitution for paraffinization. The tissues were embedded in paraffin blocks, and sections (4 μm) between the L3 and L5 positions were prepared using a microtome (Retoratome REM-710, YAMATO, Japan). Paraffin-embedded sections were stained with H&E and LFB (MUTO PURE CHEMICALS.CO., LTD, Japan). The sections were photographed using a NanoZoomer-SQ (Hamamatsu Photonics K.K., Shizuoka, Japan).

Mice were euthanized by cervical dislocation, and ear specimens were cut off at 7 days after imiquimod application. The mouse ear specimens were fixed in a 10 % formalin solution for 24 hr. The ears were dehydrated through the use of alcohol, followed by xylene, and then embedded in paraffin blocks as described above. Five-micrometer paraffin sections were stained with H&E. Epidermal thickness was determined by measuring three points per ear tissue with NanoZoomer-SQ. The average value was used to calculate the thickness of the epidermis of a mouse.

#### Anti-MOG_35-55_ antibody measurement after PTX administration

Mice were intraperitoneally injected with PTX (200 ng per mouse) at days 0 and 2. On day 30 after the first PTX injection, whole-blood samples were collected via cardiac puncture under anesthesia. The blood samples were stored at 4°C overnight, and sera were collected by centrifugation at 3000 rpm for 10 min. MOG_35-55_ peptides (1 μg/well of a 96-well plate in PBS) were coated on a 96-well plate at 4°C overnight. The wells were blocked with 10% FBS in PBS at room temperature for 1 hr. Serum samples were diluted 100-fold, and 50 μl of the diluted samples were incubated at 4°C overnight. HRP-conjugated goat anti-mouse IgG (0.8 μg/ml) (Jackson ImmunoResearch, West Grove, PA, USA) or HRP-conjugated goat anti-mouse IgG1, IgG2a, IgG2b, and IgG3 (Proteintech Japan, Tokyo, Japan) were added and incubated at room temperature for 1 hr. Then, a 3, 3′, 5, 5′-tetramethylbenzidine solution (DAKO, Agilent Technologies, CA, USA) for IgG was added as the substrate, and 1 N HCl was used to stop the color development. Antibody concentrations were determined by measuring the absorbance at 450 nm for IgG and the other subclasses.

#### Histological encephalomyelitic severity evaluation

Three tissue sections were used to evaluate the severity of spinal cord inflammation. Encephalomyelitic severity was determined following the criteria used in the previous report. The requirements for encephalomyelitic severity were as follows: 0, no inflammation; 1, cellular infiltration only in the perivascular areas and meninges; 2, mild cellular infiltration in the parenchyma (inflammatory cells infiltrating less than one-third of the white matter area); 3, moderate cellular infiltration in the parenchyma (inflammatory cells infiltrating more than one-third of the white matter area); and 4, severe cellular infiltration in the parenchyma. Demyelination conditions were assessed by LFB staining, using the following criteria: 0, no demyelination; 1, mild demyelination; 2, moderate demyelination; 3, severe demyelination.^10^^,^^43^

#### Stimulation of TLR agonists and U11snRNA

GM-DCs induced from bone marrow cells with GM-CSF (20 ng/ml) were harvested using Cell Dissociation Solution Non-enzymatic 1× (Sigma-Aldrich) and resuspended in R10. GM-DCs were seeded at 1 × 10^4^ cells per well in a 96-well plate and stimulated with TLR agonists for 72 hr. Poly(I:C) (1 μg/ml), LPS (1 ng/ml), imiquimod (1 μg/ml), and ODN1669 (0.1 μM) were used as agonists for TLR3, TLR4, TLR7, and TLR9, respectively. For U11snRNA stimulation, Lipofectamine 3000, a cationic lipid, was used for RNA delivery (ThermoFisher), and the mixture of Lipofectamine reagents and U11snRNA was prepared according to the manufacturer’s protocol. Splenocytes were seeded at 4 × 10^5^ cells per well in a 96-well plate and stimulated with 0.5 and 1 μg/ml of U11snRNA for 72 hr. The supernatants were stored at -80°C until use.

#### RNA extraction from murine and human sera

Whole blood samples of mice and humans were collected by cardiac puncture under anesthesia. The blood samples were left to clot overnight at 4°C, then centrifuged at 3,000 rpm for 10 min to separate the samples from the coagulated blood. The sera were collected and stored at –80°C until assayed. *eGFP* mRNA fragment described in Table S2 was added to murine and human sera as an external control (the final concentration is 1167 ng/ml), and RNA in sera was extracted using NucleoSpin miRNA Plasma (TAKARA bio, Japan). Total RNA was quantified with NanoDrop (ThermoFisher).

#### Serum detection of U11snRNA

Serum RNA was reverse-transcribed using the PrimeScript RT reagent kit with gDNA Eraser (TAKARA bio). For the analysis of U11snRNA, the *Rnu11* reverse primer was used for transcription. The quantitative PCR was performed in a total reaction volume of 10 μl, including 5 ng of total RNA equivalent, SYBR Green I (TAKARA bio), and a set of forward and reverse primers (ThermoFisher) in triplicate samples. Quantification was performed on the CFX384^TM^ Real-Time system (Bio-Rad Laboratories, Inc., Hercules, CA, USA). The cycling program was 1 cycle of 95°C for 1 min, following 44 cycles of a set of 95°C for 3 s and 60°C for 30 s. The primer sets used to detect each transcript in U11snRNA, U1snRNA, and eGFP are described in Table S1. The final concentration of the primers was 400 nM. The target genes and the external control gene were quantified simultaneously in one plate, with water samples as the negative reference. The difference in RNA quality and initial quantity of samples was normalized to eGFP (Table S2). The relative expression of the targeted gene (2ˆ-ddCt method) was calculated from the following equation: 2ˆ-dCt, where dCt = average Ct for the gene of interest – average Ct for the housekeeping gene. The fold change of a targeted gene on days 3 and 4 relative to a sample on day 2 was determined by the following: 2ˆ-ddCt, where ddCt = (average Ct for gene of interest – average Ct for the gene of a housekeeping gene) each sample on days 3 and 4 – (average Ct for gene of interest – average Ct for the housekeeping gene) sample on day 2. The standard deviation was calculated according to the mathematical method described in earlier reports.^44^^,^^45^

#### Homology searching of the intracellular domain of human DCIR

The amino acid sequence of the intracellular domain (60 amino acids from methionine to alanine) of human DCIR was analyzed with FASTA Search. Multiple sequence alignments of the resulting top 20 sequences were conducted with Clustal W.

### Quantification and statistical analysis

Statistical analyses were performed using GraphPad Prism 9.0 software. The statistical significance between groups was determined using the two-tailed unpaired Students’ *t*-test (∗*P*<0.05; ∗∗*P*<0.01; ∗∗∗*P*<0.001; NS, not significant, in the paper). The clinical scores of EAE were evaluated using the Mann-Whitney U test or ANOVA with Tukey HSD post hoc test, and the incidence of EAE was analyzed using the χ2 test. The difference between groups was considered statistically significant at *P*<0.05.
