# Supplementary material for: DCIR-mediated inhibitory regulation of TLR7-MyD88 axis prevents autoimmune neuroinflammation
Source: iScience. 2026 May 29;29(6):116094. doi: 10.1016/j.isci.2026.116094 (PMC13241762; doi:10.1016/j.isci.2026.116094)
Supplement: Document S1. Figures S1–S6 and Tables S1–S3 [file mmc1.pdf]

iScience, Volume *29*

Supplemental information

DCIR-mediated inhibitory regulation of TLR7-MyD88 axis prevents autoimmune neuroinflammation

Atsushi Fujioka, Kenji Shimizu, Saki Nakayama, Dai Ueno, Yuki Chiba, Ichiro Nakashima, Juichi Fujimori, Yuko Shirota, Yoichiro Iwakura, Yasushi Muraki, Akira Nakamura, and Tomonori Kaifu

Supplementary Figure 1

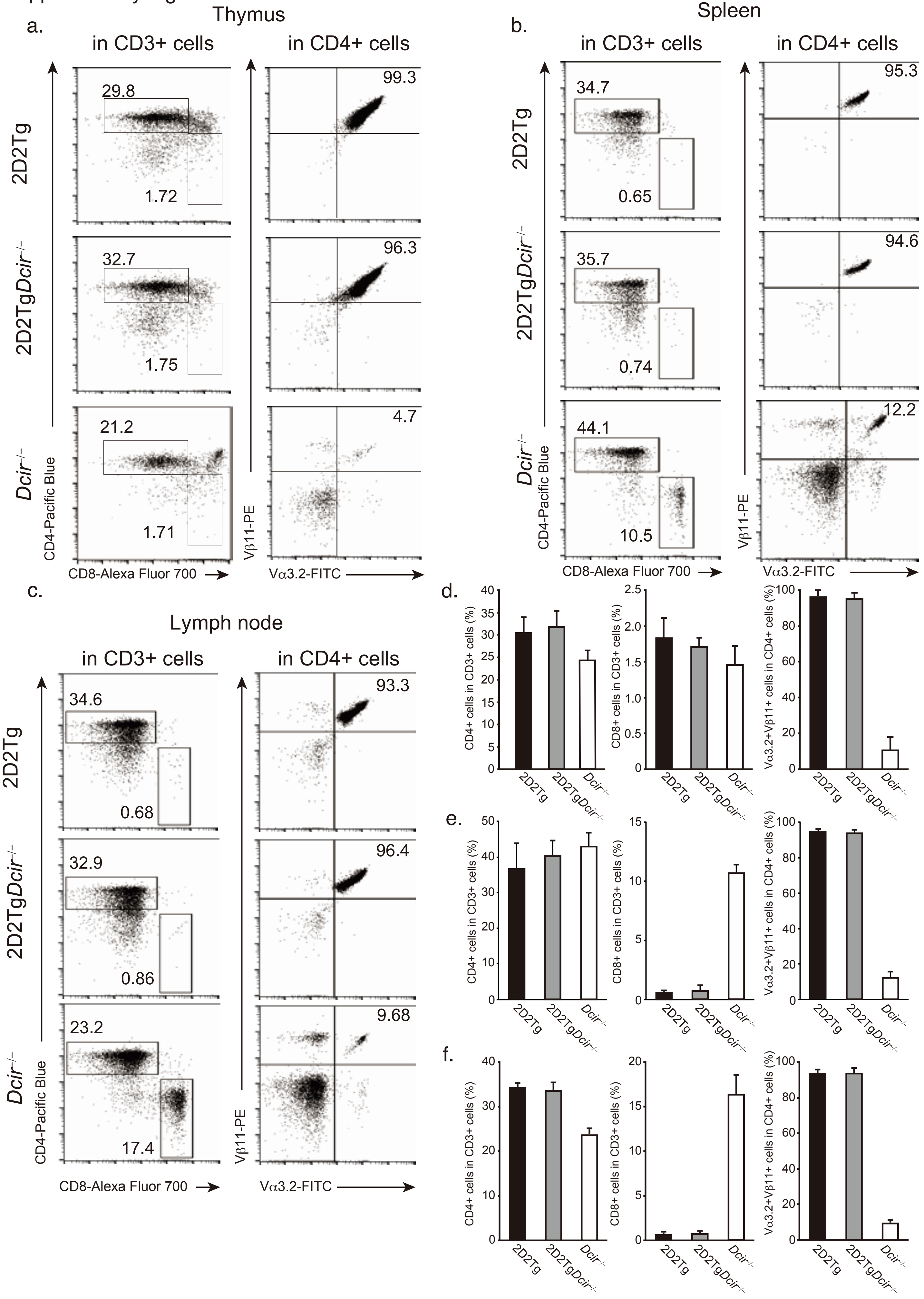

Fujioka et al.

Supplementary Figure 2

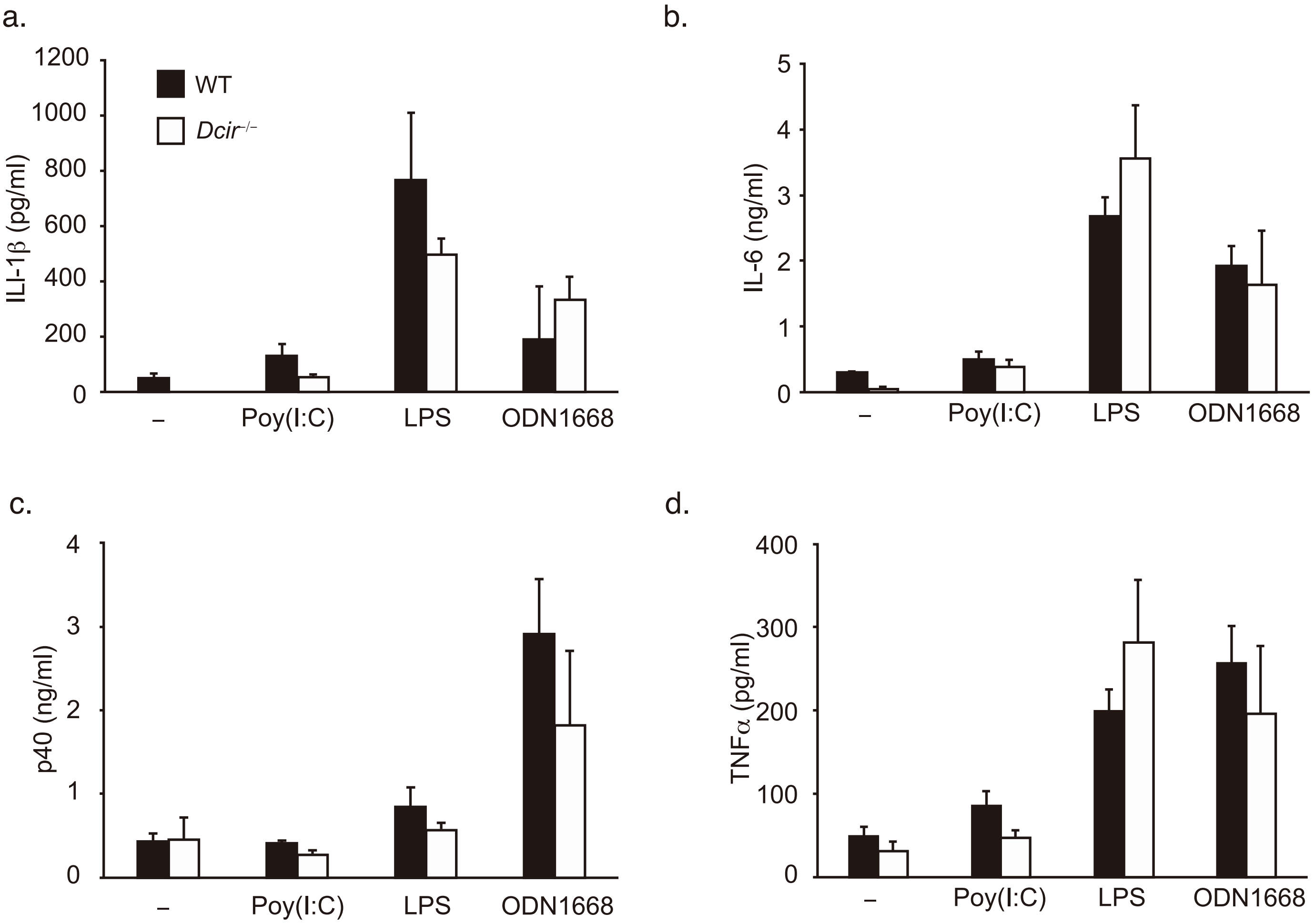

Supplementary Figure 3

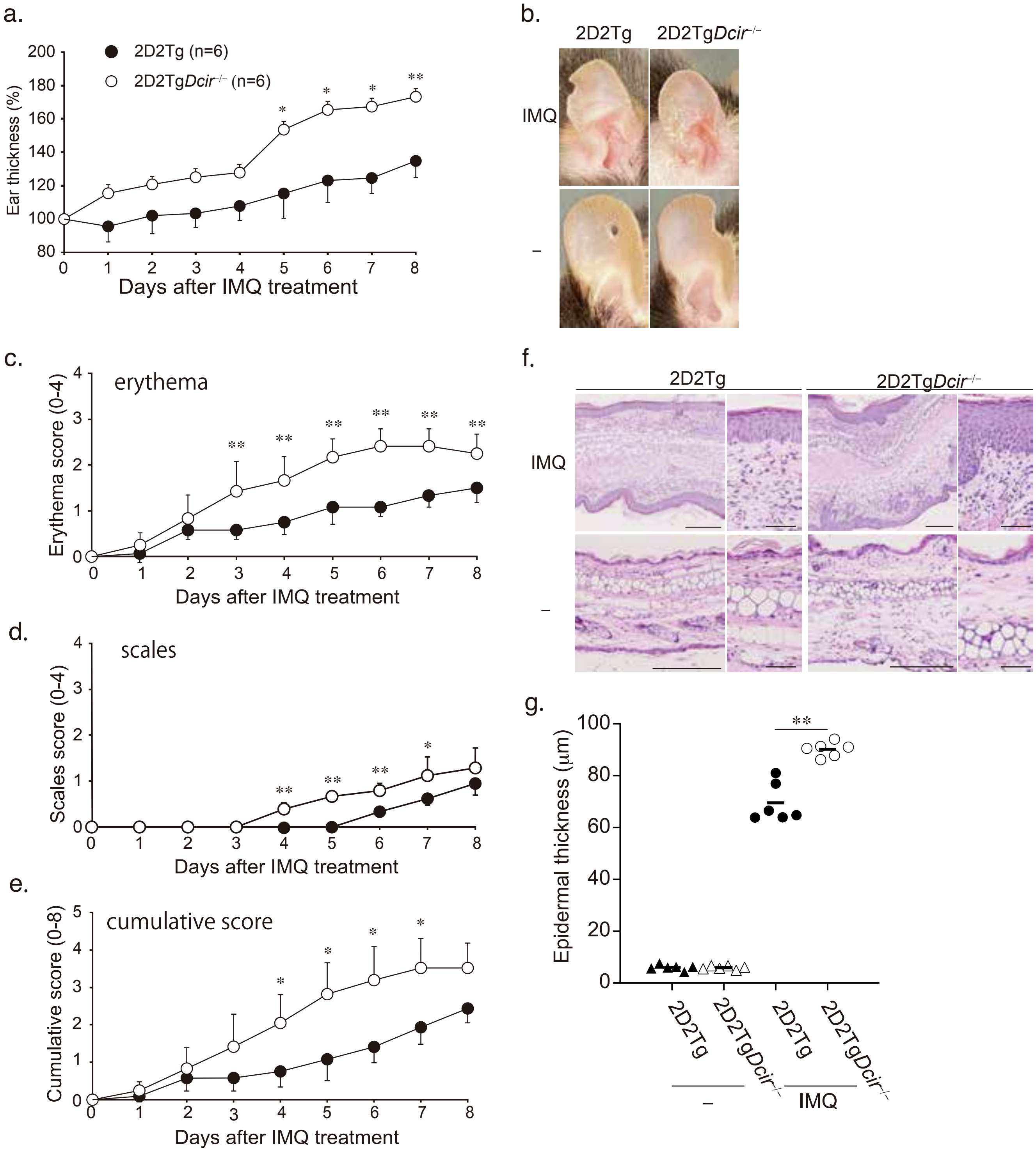

Fujioka et al.

Supplementary Figure 4

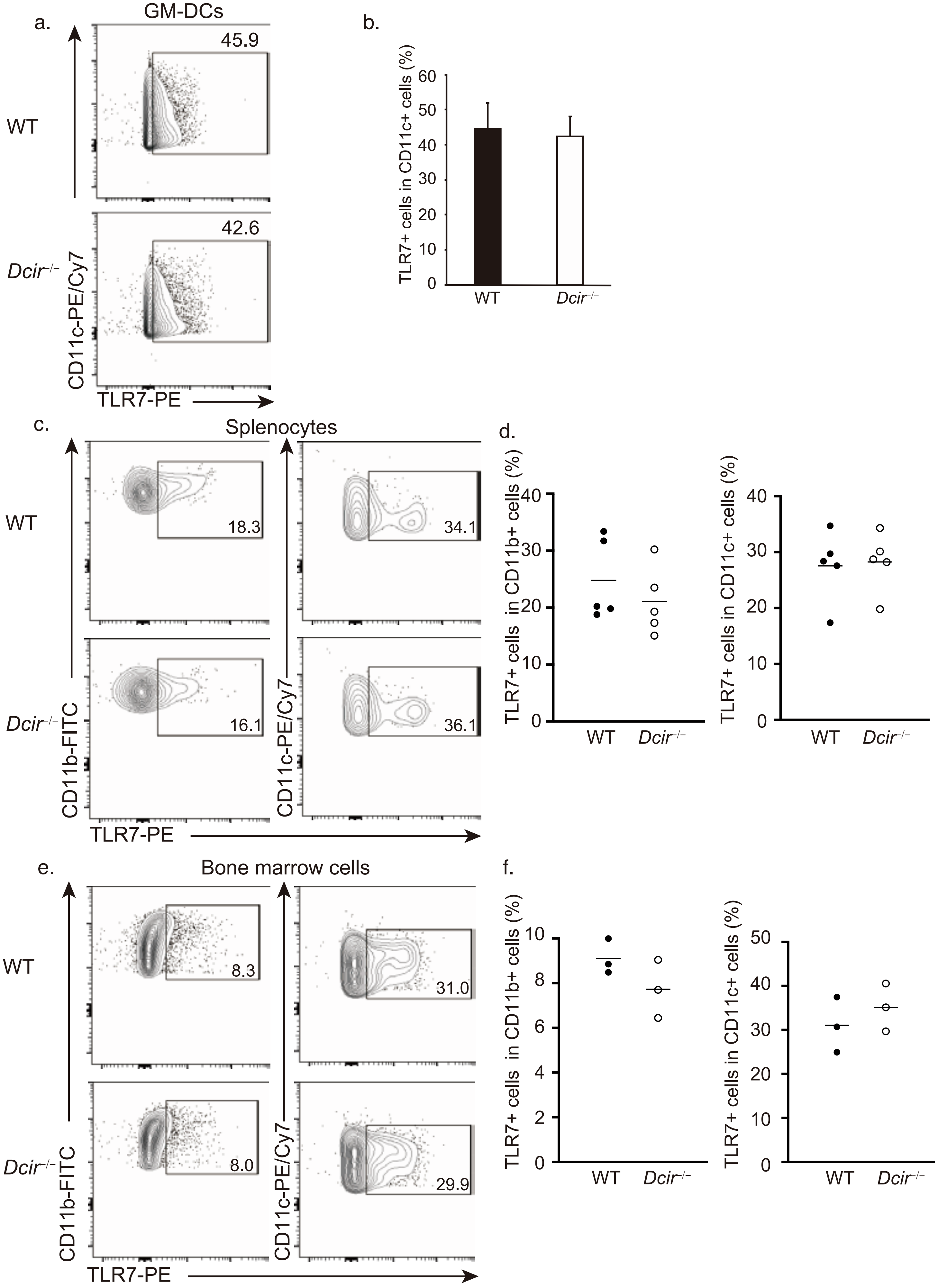

Fujioka et al.

Supplementary Figure 5

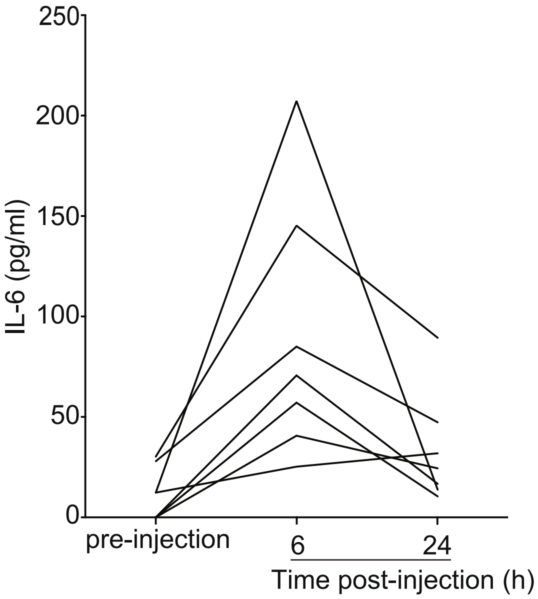

Fujioka et al.

Supplementary Figure 6

human	MTSEITYAEVRFKNEFKSSGINTASSAASKERTAPHKSNTGFPKLLCA
gelada	MTSEITYAEVRFQNESKFSGIDSASSAASKERTAPHKSNTGFSKLLCA
olive baboon	MTSEITYAEVRFQNESKFSGIDSASSAASKERTAPHKSNTGFSKLLCA
drill	MTSEITYAEVRFQNESKFSGIDSASSAASKKRTAPHKSNTGFSKLLCA
sooty mangabey	MTSEITYAEVRFQNESKFSGIDSASSAASKERTAPHKSNTGFSKLLCA
rhesus monkey	MTSEITYAEVRFQNESKFSGIDSASSAASKKRTAPHKSNTGFSKLLCA
crab-eating macaque	MTSEITYAEVRFQNESKFSGIDSASSAASKKRTAPHKSNTGFSKLLCA
Pere David’s macaque	MTSEITYAEVRFQNESKFSGIDSASSAASKKRTAPHKSNTGFSKLLCA
green monkey	MTSEITYAEVRFQNESKFSGIDSASSAASKERTAPHKSNTGFSKLLCA
black snub-nosed monkey	MTSEITYAEVRFQNESKFSGINSASSAASKERTAPHESNTGFSKLLCA
golden snub-nosed monkey	MTSEITYAEVRFQNESKFSGINSASSAASKERTAPHESNTGFSKLLCA
Angola colobus	MTSEITYAEVRFQNESKFSGINSASSAASKERTAPHESNTGFSKLLCA
western lowland gorilla	MTSEITYAEVRFKNEFKSSGINTASSAASNERTAPHKSNTGFPKLLCA
chimpanzee	MTSEITYAEVRFKNEFKSSGINTASSAASKERTAPHKSNAGFPKLPCA
bonobo	MTSEITYAEVRFKNEFESSGINTASSAASKERTAPHKSNAGFPKLPCA
sumatran orangutan	MTSEITYAEVRFKNEFKSSGINSASSAASKERTAPHKSNTGFPKLLCA
Bornean orangutan	MTSEITYAEVRFKNEFKSSGINSASSAASKERTAPHKSNTGFPKLLCA
siamang	MTSEITYAEVRFKHEFKSSGINSASSAASKERTAPHKSNTGFPKLLCA
silvery gibbon	MTSEITYAEVRFKHEFKSSGINSASSAASKEMTAPHKSNTGFPKLLCA
northern white-cheeked gibbon	MTSEITYAEVRFKHEFKSSGINSASSAASKERTAPHKSNTGFPKLLCA

Supplementary Figure1. DCIR is dispensable for the differentiation of thymocytes, splenocytes, and lymphocytes under steady conditions. Thymocytes, splenocytes, and lymphocytes were prepared from 2D2Tg mice, 2D2Tg*Dcir*$^{-/-}$ mice, and *Dcir*$^{-/-}$ mice, and the cell distribution was analyzed in a flow cytometer. A-**C**. CD4+ and CD8+ cell populations and TCR repertoires consisted of Vα3.2 and Vβ11 chains in CD4+ cells in the thymus, spleen, and lymph node under steady-state conditions. D-**F**. Histogram analysis of cell distribution of a-c. CD4+ and CD8+ cells in CD3+ cells and CD4+ cells with Vα3.2 and Vβ11 chains. Black bar: 2D2Tg mice, grey bar: 2D2Tg*Dcir*$^{-/-}$ mice, white bar: *Dcir*$^{-/-}$ mice. The numeric digits in dot plots indicate the cell proportions within rectangles and quadrats. The bars show the mean ± SD of triplicate wells and the data are representative of three independent experiments.

Supplementary Figure2. Cytokine production from GM-DCs upon stimulation with TLR3, TLR4, and TLR9 agonists wasn't affected by DCIR deficiency. GM-DCs (1×10^6 cells) were stimulated with TLRs agonists for 72 h and the amount of cytokines (**A**. IL-1β, **B**. IL-6, **C.** p40, **D.** TNFα**)** was determined with ELISA. TLRs agonists: Poly(I:C) stimulation (10 ng/ml); LPS stimulation (10 ng/ml); ODN1668 stimulation (0.1 μM). Black column; WT GM-DCs, white column; *Dcir*$^{-/-}$ GM-DCs. Data is shown as mean ± SD of triplicate wells. These data are representative of the three independent experiments.

Supplementary Figure3. DCIR regulates the TLR7 signal in IMQ-induced skin inflammation. A. IMQ-induced skin inflammation in 2D2Tg*Dcir*$^{-/-}$ mice. Evaluation of IMG-applied ears from day 0 to 8 (2D2Tg*Dcir*$^{-/-}$ mice; n=6, 2D2Tg mice; n=6). Ears were treated with IMQ cream administered intramuscularly (IM) daily for 8 days, and ear thickness was measured daily using a microcaliper. Ear thickness is shown as the percentage relative to the ear thickness at day 0. The bars show the mean ± SEM. Statistical significances were evaluated using a two-tailed unpaired Student's *t*-test (*, $P<0.05$). B**.** Ear images of IMQ-induced skin inflammation of 2D2Tg mice and 2D2Tg*Dcir*$^{-/-}$ mice at day 8. C**-E.** Clinical assessments of ears of 2D2Tg mice and 2D2Tg*Dcir*$^{-/-}$ mice after IMQ cream treatment (c. redness, d. scales, and e. cumulative score). Data are the mean ± SEM. Statistical significances were evaluated using the Mann-Whitney *U* test (*, $P<0.05$, **, $P<0.01$). F**.** Ear sections of 2D2Tg mice and 2D2Tg*Dcir*$^{-/-}$ mice at day 8 with or without IMQ cream were stained with H&E and one of the representative sections is shown (scale bar, 250 μm in low-power fields and 50 μm in high-power fields). **G**. Epidermal thickness was measured by NanoZoomer-SQ in H&E staining sections. The data are representative of two independent experiments. Statistical significances were evaluated using one-way ANOVA with Tukey post hoc test (**, $P<0.01$).

Supplementary Figure4. TLR7 expression is comparable between WT and *Dcir*$^{-/-}$ GM-DCs, splenocytes, and BM cells. A. TLR7 expression in GM-DCs. GMDCs from WT and *Dcir*$^{-/-}$ mice were stained with antibodies against CD11c and then intracellularly

stained with antibodies against TLR7. B**.** Histogram analysis of TLR7-positive cells. Black column; WT GM-DCs, white column; *Dcir*$^{-/-}$ GM-DCs. Data is shown as mean ± SD of triplicate wells. These data are representative of the three independent experiments. TLR7-positive cells in CD11b+ and CD11c+ splenocytes and bone marrow cells (C **and F**) from WT (n=5) and *Dcir*$^{-/-}$ (n=5) mice were detected using a flow cytometer. CD3-negative splenocytes were plotted with CD11b and CD11c, and TLR7+ cells in CD11b+ and CD11c+ cells were determined. The numeric digits in dot plots present the percentage of cell proportion inside rectangles. Histogram analysis of TLR7-positive cells in splenocytes (**D**) and bone marrow cells (**F**). Each circle represents one mouse. The black circle is WT mouse, and the white circle is *Dcir*$^{-/-}$ mice. The horizontal bars show the mean of five mice, and the data are representative of two independent experiments.

Supplementary Figure5. PTX-treatment increases serum IL-6. Blood samples were prepared from 2D2Tg mice (n=7) at 3 days before injection, 6 hr and 24 hr after injection. Serum IL-6 concentration was quantified by ELISA. Each line presents an individual mouse.

Supplementary Figure6. Histidine and glycine in the intracellular domain of DCIR are conserved across different primate species. Amino acid sequence alignment of the intracellular domain of DCIR. The GWAS data on MS severity were available from IMSGC. A homology search of the human DCIR intracellular domain, containing the rs2024301 SNV and the rs117213717 SNV, was conducted using the FASTA program.

The top 20 species were listed. A single black circle with a rectangular box indicates the amino acid that the rs2024301 SNV changes into leucine. Double black circles with a rectangular box display the amino acid rs117213717 SNV changes into arginine.

Supplemental Table S1. PCR primer sets

Gene	Forward (5' to 3')	Reverse (5' to 3')
**qPCR**		
Rnu11 (mouse)	CGGAATCGACATCAAGAGATTT	AACGATCACCAGCTGCC
Rnu11 (human)	CGGAATCGACATCAAGAGATTT	AACGATCACCAGCTGCC
Rnu1 (mouse)	GATACCATGATCACGAAGGTGGTT	CACAAATTATGCAGTCGAGTTT
EGFP	GTACAACTACAACAGCCACAACG	TAGTGGTCGGCGAGCTGCAC

Supplemental Table S2. RNA sequences

Murine U11snRNA (5' to 3')
AAAAAGGGCUUCUGUCGUGAGUGGCACACGCAGGGCAACUCGAUUGCUGUGCGUGCG GAAUCGACAUCAAGAGAUUUCGGAAGCAUAAUUUUUUGGUAAUUGGGCAGCUGGUGA UCGUUGGUCCCGGCGCCCUU
eGFP mRNA (5' to 3')
UAGUGGUCGGCGAGCUGCACGCUGCCGUCCUCGAUGUUGUGGCGGAUCUUGAAGUU CACCUUGAUGCCGUUCUUCUGCUUGUCGGCCAUGAUAUAGACGUUGUGGCUGUUGU AGUUGUAC

Supplemental Table S3. List of antibodies

Antibodies	Label	Clone	Source	Catlog #	Identifier
For FACS analysis					
TCR repertoire					
Anti-mouse CD3	PE/eFluor610	17A2	eBiosciencce	61-0032-82	AB_2815286
Anti-mouse CD4	Pacific blue	RM4-5	BioLegend	100531	AB_493374
Anti-mouse CD8	AlexaFluro700	53-6.7	BioLegend	100729	AB_493702
Anti-mouse Vα3.2	FITC	RR3-16	BioLegend	135403	AB_1937236
Anti-mouse Vβ11	PE	RR3-15	BioLegend	139003	AB_10613472
Intracellular staining					
Anti-mouse CD3	PE/Cy5	145-2C11	BioLegend	100310	AB_312675
Anti-mouse CD4	FITC	GK1.5	BD Biosciences	553729	AB_395013
Anti-mouse TNF-α	PE	MP6-XT22	BioLegend	506305	AB_315426
Anti-mouse IL-17	PE/Cy7	TC11-18H10.1	BioLegend	506922	AB_2125010
Anti-mouse IFN-γ	APC/Cy7	XMG1.2	BioLegend	505849	AB_2616697
Anti-mouse Ki67	APC	16A8	BioLegend	652405	AB_2561929
TLR7 staining					
Anti-mouse TLR7	PE	A94B10	BioLegend	160003	AB_2860749
Anti-mouse CD11b	FITC	M1/70	BioLegend	101206	AB_312789
Anti-mouse CD11c	PE/Cy7	N418	BioLegend	117317	AB_493569
Infiltration cells staining					
Anti-mouse CD11b	APC/Cy7	M1/70	BioLegend	101226	AB_830642
Anti-mouse CD11c	FITC	N418	BioLegend	117305	AB_313774
Anti-mouse CD3	PE/Cy5	145-2C11	BioLegend	100310	AB_312675
Anti-mouse B220	AlexaFluor700	RA3-6B2	BioLegend	103231	AB_493716
Anti-mouse CD4	PE/Cy7	RM4-5	BioLegend	100527	AB_312728
Anti-mouse CD8	APC	53-6.7	BioLegend	100711	AB_312750
